# Impact of Contrast-Enhanced Mammography on Breast Imaging Reporting and Data System (BI-RADS) Reclassification: Correlation With Histopathology and Clinical Outcomes

**DOI:** 10.7759/cureus.100352

**Published:** 2025-12-29

**Authors:** Mariam Malik, Umal Baneen Zahra, Faisal Ehsan Cheema, Aamena Irfan Shami, Amira Shami, Muhammad Imran

**Affiliations:** 1 Radiology, Institute of Nuclear Medicine and Oncology (INMOL) Cancer Hospital, Lahore, PAK; 2 Radiology, Islamabad Diagnostic Center, Islamabad, PAK; 3 Oncology, Institute of Nuclear Medicine and Oncology (INMOL) Cancer Hospital, Lahore, PAK; 4 Pathology, Allama Iqbal Medical College, Lahore, PAK

**Keywords:** benign, contrast enhanced mammography, digital mammography, malignant, bi-rads

## Abstract

Purpose: This study aims to evaluate how contrast-enhanced mammography (CEM) modifies the Breast Imaging Reporting and Data System (BI-RADS) classification initially assigned on digital mammography (DM), and to assess the accuracy of CEM-driven upgrades, downgrades, and confirmations using histopathology as reference.

Methods: This was a retrospective observational study that included symptomatic patients who underwent both DM and subsequent CEM within a short interval. The initial BI-RADS grading was assigned based on DM findings, while CEM findings were categorized as either correct or incorrect upgrades, downgrades, or confirmations, based on final histopathology or follow-up results. The study included 60 patients across all grades of BI-RADS.

Results: Among patients initially assigned BI-RADS 0-5 on DM, CEM correctly upgraded 12 lesions and correctly downgraded six lesions. CEM also incorrectly upgraded seven lesions and incorrectly downgraded two lesions. The BI-RADS category was correctly confirmed in the remaining 33 patients. The lesions upgraded in several BI-RADS 0 and 4 cases were occult or subtle enhancement revealed as invasive carcinoma on histopathology. False-positive upgrades were seen in cases of fibroadenoma and sclerosing adenosis, while false-negative downgrades occurred in two BI-RADS 5 lesions that showed no enhancement but proved malignant on histopathology. CEM also aided in downgrading post-surgical and inflammatory changes accurately, improving confidence in excluding recurrence or malignancy.

Conclusion:* *CEM improves the diagnostic performance of digital mammography by providing additional functional information related to tumor vascularity, allowing more accurate characterization of breast lesions. In this study, CEM correctly upgraded, downgraded, or confirmed BI-RADS classifications in the majority of patients, particularly in cases with dense breast tissue and indeterminate mammographic findings. By revealing mammographically occult malignancies and confidently excluding benign or post-treatment changes, CEM reduced diagnostic uncertainty and supported more appropriate patient management. Although false-positive and false-negative findings were observed, these limitations underscore the need for careful interpretation alongside conventional imaging and clinical correlation.

## Introduction

Digital mammography (DM) remains the cornerstone of breast cancer screening and diagnosis. However, its diagnostic sensitivity is significantly reduced in dense breasts and in cases with overlapping fibroglandular tissue [1,2]. The introduction of contrast-enhanced mammography (CEM) brought forth an adjunct functional imaging tool that combines anatomical and vascular assessment in a single examination [3]. 

CEM has shown promise in detecting angiogenic activity associated with malignancy, similar to magnetic resonance imaging (MRI) [4]. Despite this, its role in redefining Breast Imaging Reporting and Data System (BI-RADS) categorization across different mammographic findings remains underexplored, particularly in routine clinical settings where CEM follows an inconclusive or indeterminate DM.

The primary objective of this study was to evaluate the impact of CEM on BI-RADS reclassification compared with DM, using histopathology or imaging follow-up as the reference standard. Secondary objectives included assessment of imaging-pathology concordance, evaluation of diagnostic performance metrics (sensitivity, specificity, positive predictive value (PPV), negative predictive value (NPV), and accuracy), and analysis of CEM performance across different breast density categories. The study focused on diagnostic refinement rather than direct assessment of downstream clinical management outcomes.

## Materials and methods

Study design and population

This retrospective observational study included symptomatic women who underwent CEM between May 1, 2025, and July 1, 2025. This period represents the CEM imaging acquisition window only. Patients referred for CEM following inconclusive or indeterminate findings on DM, dense breast parenchyma (ACR C or D), palpable lumps with negative DM, or post-treatment evaluation during the study period were included. Patients with incomplete records, known contrast allergies, or contraindications to iodinated contrast were excluded. The study was approved by the Institutional Review Board/Ethics Committee of Atomic Energy Cancer Hospital Institute of Nuclear Medicine and Oncology (INMOL), Lahore, Pakistan (approval number: INMOL-188-(08)), and all procedures followed standard radiological safety protocols.

Data collection

Clinical outcomes, including histopathological correlation, postoperative assessment, chemotherapy response evaluation, and imaging follow-up of ≥6 months for benign lesions, were obtained retrospectively from institutional medical records and prior imaging performed before and after the CEM examination.

Imaging technique

DM and CEM were performed using Selenia® Dimensions® Mammography System (Hologic, Inc., Marlborough, Massachusetts, United States) in craniocaudal (CC) and mediolateral oblique (MLO) views. CEM was conducted on the same or subsequent day using a dual-energy acquisition protocol. Intravenous iodinated contrast material (Omnipaque) 300 mg I/mL was injected with a dose of 1.5 mL/kg. The contrast was given at a rate of 3 mL/s via power injector, followed by a 30 mL saline flush. Acquisition commenced two minutes post injection, and the total acquisition time was five to seven minutes. Both low-energy (28 - 32 kVp) and high-energy (45-49 kVp) images were obtained for recombination using the manufacturer’s dual-energy algorithm. No delayed views were obtained.

Quality Control

Inter departmental quality control was performed weekly and included several parameters like C-Arm Gantry check (Source-to-Image Distance (SID) indicator marks, angulation indicator, locks, collimator light, smoothness of motion, grid function, compression device function, compression thickness display, compression force display), acquistion work check (glass shield, exposure switches, power controls, monitors, technique charts) and accessories check (foot pedals, compression paddles clean and not cracked, face shields clean and not cracked, disinfection materials available).

Image Interpretation

All examinations were independently interpreted by two radiologists with more than five years of experience in breast imaging. Readers were blinded to histopathology and clinical outcomes at the time of interpretation. Inter-observer agreement for BI-RADS categorization was assessed using Cohen’s kappa (κ) statistic. For continuous variables, including lesion size measurements on CEM, inter-observer reliability was evaluated using the intraclass correlation coefficient (ICC). Discrepancies in BI-RADS categorization were subsequently resolved by consensus for final analysis.

Digital mammography: Each DM study was initially assigned a BI-RADS category (0-5) according to the American College of Radiology (ACR) BI-RADS 5th edition lexicon [5].

Contrast-enhanced mammography:* *CEM studies were interpreted independently. Lesions were assessed for morphology, enhancement pattern, and intensity on recombined images. Each CEM finding was assigned a corresponding BI-RADS category, which was then compared to the initial DM-based BI-RADS.

BI-RADS classification (DM and CEM)

All breast findings were evaluated and categorized according to the ACR BI-RADS Atlas, applying standardized descriptors for both DM and CEM. BI-RADS provides uniform terminology, malignancy-risk stratification, and management recommendations across imaging modalities.

For DM, lesion characterization included assessment of mass shape and margins, architectural distortion, asymmetries, and calcification morphology/distribution. For CEM, lesions were evaluated on both the low-energy (LE) images, equivalent to standard mammography, and the recombined contrast-enhanced images, which depict enhancement patterns reflecting vascularity.

Findings were assigned BI-RADS categories as follows: (i) BI-RADS 0: Incomplete evaluation needing additional mammographic views, targeted ultrasound, or further diagnostic workup; (ii) BI-RADS 1: Negative exam with no structural abnormality or enhancement; (iii) BI-RADS 2: Benign findings such as simple cysts, benign calcifications, or non-enhancing benign masses; (iv) BI-RADS 3: Probably benign findings with <2% risk of malignancy; typically well-circumscribed masses or faint/minimal enhancement prompting short-interval follow-up; (v) BI-RADS 4: Suspicious abnormalities based on morphology (on DM) or moderate/irregular enhancement (on CEM); biopsy recommended. Subcategories (4A-4C) reflected increasing suspicion; (vi) BI-RADS 5: Highly suspicious lesions showing spiculated margins, malignant calcification patterns, or intense/heterogeneous enhancement consistent with ≥95% likelihood of malignancy; (v) BI-RADS 6: Proven malignancy on histopathology demonstrating corresponding imaging features.

In this study, BI-RADS assessment was performed by correlating mammographic morphology with CEM enhancement characteristics to assign the most accurate final category based on the latest ACR BI-RADS Atlas criteria.

Lesions classified as BI-RADS 6, representing biopsy-proven malignancy, were not subjected to upgrade or downgrade analysis. These cases were included solely to assess concordance between imaging findings and histopathology and were excluded from diagnostic reclassification analyses.

Classification of CEM Outcomes

CEM findings were grouped into five categories based on final histopathology or follow-up: (i) Correct Upgrade:Higher BI-RADS on CEM confirmed malignant; (ii) Incorrect(False-Positive) Upgrade:Higher BI-RADS on CEM but benign on histopathology; (iii) Correct Downgrade: Lower BI-RADS on CEM confirmed benign; (iv) Incorrect (False-Negative) Downgrade:* *Lower BI-RADS on CEM but malignant on histopathology; (v) Confirmation: Same BI-RADS category on both DM and CEM with concordant final diagnosis.

Reference standard and statistical analysis

Histopathology served as the reference standard in lesions that underwent biopsy or surgical excision. In cases where a biopsy was not performed, lesion status was determined based on stable imaging findings on follow-up examinations of at least six months’ duration. Of the total lesions included, 27 were confirmed by histopathology, and 33 were confirmed by imaging follow-up of at least six months’ duration.

Data was analysed using IBM SPSS Statistics for Windows, version 27 (IBM Corp., Armonk, New York, United States). Descriptive statistics were used to calculate frequencies and percentages of CEM’s impact on BI-RADS classification. Diagnostic performance parameters (accuracy, sensitivity, specificity, PPV, and NPV) were computed where applicable. Specificity was calculated using lesions ultimately confirmed as benign by histopathology or adequate imaging follow-up. True-negative cases included lesions correctly categorized as benign on CEM. BI-RADS 3 lesions and follow-up-only confirmation cases were included in specificity calculations only when the final benign status was established. Diagnostic accuracy was calculated by considering true-positive, true-negative, and correctly confirmed cases in which CEM-based BI-RADS categorization was concordant with the reference standard. Accuracy was defined as: (True Positives + True Negatives + Correctly Confirmed Cases)/Total Number of Lesions.

All statistical analyses were conducted on a per-patient basis. In patients with multiple lesions, the lesion with the highest BI-RADS category or the lesion that determined clinical management was considered the index lesion for analysis. Diagnostic performance metrics, including sensitivity, specificity, positive predictive value, and negative predictive value, were calculated using patient-level outcomes to avoid assumptions of independence associated with lesion-based analysis. 

Inter-observer agreement for BI-RADS categorization was quantified using Cohen’s kappa (κ), interpreted according to Landis and Koch criteria. Inter-observer reliability for continuous variables was assessed using the intraclass correlation coefficient (ICC) with a two-way random-effects model and absolute agreement definition. κ and ICC values were reported alongside 95% confidence intervals (CIs).

## Results

Detailed observations

Upgraded Lesions (n=19)

CEM upgraded 19 cases in total, 12 correctly (true-positive malignancies) and seven incorrectly (false positives). Correct upgrades predominantly occurred in dense breasts (initial BI-RADS 0-3), revealing previously occult invasive ductal and lobular carcinomas. False-positive upgrades included fibroadenomas, papillomas, and sclerosing adenosis, which showed enhancement patterns mimicking malignancy.

Downgraded Lesions (n=8)

Of these, six were correctly downgraded to benign (e.g., post-surgical scars, post-chemotherapy response assessment mammograms, mastitis, cysts), while two were incorrectly downgraded (false negatives), both representing non-enhancing invasive ductal carcinoma with low vascularity.

Confirmed Lesions (n=33)

The majority of cases showed concordant interpretation on both DM and CEM, including normal, benign, and malignant lesions where enhancement patterns and morphology were consistent.

Diagnostic Accuracy

CEM in this cohort demonstrated high sensitivity (85.7%) and overall diagnostic accuracy (85%), confirming its strong ability to detect malignancies that were missed or indeterminate on digital mammography. The specificity (46.2%) was modest, likely reflecting benign lesions showing enhancement (false positives) such as fibroadenomas or sclerosing adenosis, a well-documented limitation in the literature. Correctly confirmed cases represented lesions in which CEM maintained appropriate BI-RADS categorization without misclassification relative to the reference standard. No BI-RADS 6 lesions were included in the upgrade or downgrade analysis. Table 1 shows the diagnostic performance of contrast-enhanced mammography. 

**Table 1:** Diagnostic performance of contrast-enhanced mammography (CEM) compared with digital mammography (DM) Sensitivity and specificity were derived using biopsy-proven benign lesions as true negatives and malignant lesions as true positives. Modest specificity reflects enhancement in benign entities such as fibroadenomas and sclerosing adenosis, consistent with prior literature.

Parameter	Value
Diagnostic Category, n (%)	
Correct (True Positive) Upgrade	12 (20.0)
Correct (True Negative) Downgrade	6 (10.0)
Incorrect (False Positive) Upgrade	7 (11.7)
Incorrect (False Negative) Downgrade	2 (3.3)
Correct Confirmation	33 (55.0)
Total	60 (100)
Diagnostic Performance Metrics, %	
Sensitivity	85.7%
Specificity	46.2%
Positive Predictive Value (PPV)	63.1%
Negative Predictive Value (NPV)	75.0%
Overall Accuracy	85.0%

Inter-observer agreement

Inter-observer agreement for BI-RADS categorization demonstrated substantial agreement, with a Cohen’s κ value of 0.78 (95% CI: 0.69-0.86). Inter-observer reliability for lesion size measurements on CEM was excellent, with an ICC of 0.89 (95% CI: 0.82-0.94).

Representative imaging findings

**Figure 1:**
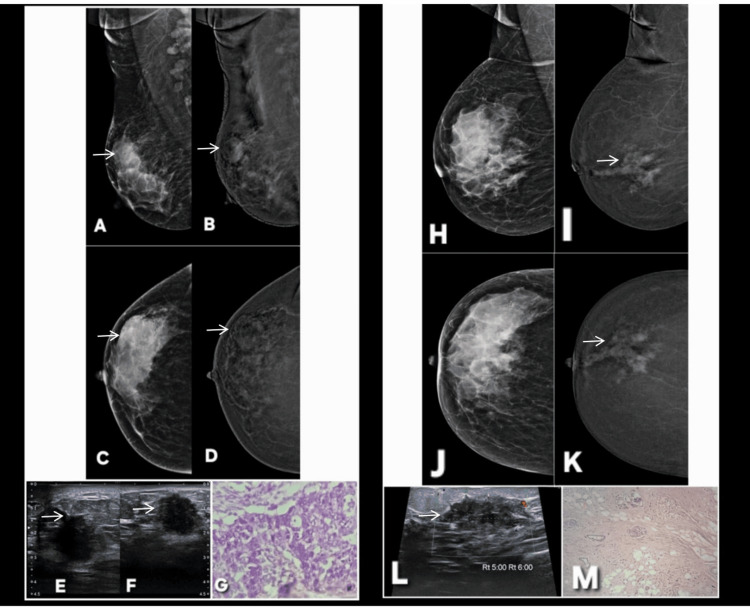
Correct upgrade of lesions on contrast-enhanced mammography (CEM). Lesion is annotated by white arrows in all radiological images. Patient π (Panels A–G): A 45-year-old female patient presented with a palpable lump in the right breast. Digital mammography (A: MLO, C: CC) demonstrates dense parenchyma, resulting in a BI-RADS 0 assessment due to near-complete obscuration of a subtle abnormality in the upper outer quadrant. Corresponding CEM views (B: MLO, D: CC) reveal a distinct enhancing mass with a central non-enhancing necrotic core, allowing definitive lesion visualization and appropriate upgrade to BI-RADS 4. Targeted gray-scale ultrasound (E, F) shows an irregular solid component with indistinct margins, concordant with malignancy. Histopathology (G) confirms invasive breast carcinoma, validating the CEM-driven upgrade. Patient ω (Panels H–M): Screening digital mammography of an asymptomatic 46-year-old female patient (H: MLO, J: CC) with a positive family history again shows dense breast tissue, with no discernible abnormality, resulting in BI-RADS 0. CEM (I: MLO, K: CC) clearly depicts a solid enhancing lesion involving dilated ducts in the central and lower outer quadrant, prompting a correct upgrade to BI-RADS 4. Targeted ultrasound (L) demonstrates a solid intraductal component with internal vascularity on Doppler assessment. Histopathology (M) confirms invasive breast carcinoma, reinforcing the diagnostic value of CEM in dense breasts. MLO: mediolateral oblique; CC: craniocaudal; BI-RADS: Breast Imaging Reporting and Data System; CEM: contrast-enhanced mammography

**Figure 2:**
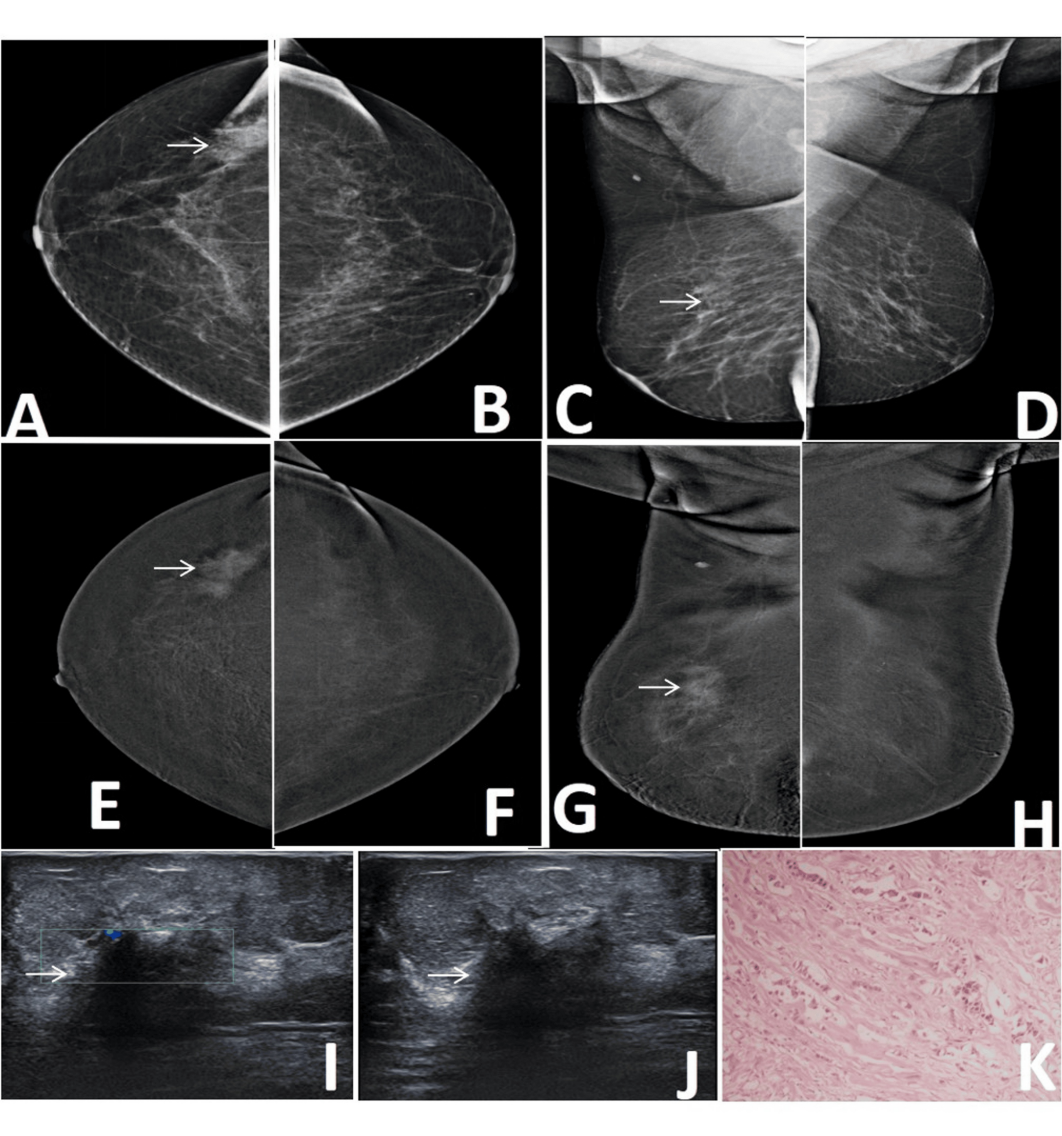
Correct upgrade of a BI-RADS 0 lesion on CEM. Lesion is annotated by white arrows in all radiological images. A 51-year-old female patient with pain in the right breast. Digital mammography (DM) of the affected breast (A: CC, C: MLO) demonstrates a focal asymmetry in the upper outer quadrant, which remains inconclusive due to the lack of any formed abnormality. Comparison with the contralateral normal breast (B: CC, D: MLO) confirms this finding as a true asymmetry, resulting in a BI-RADS 0 assessment on mammography. Contrast-enhanced mammography (E: CC, G: MLO) of the same breast reveals definite enhancement within the area of asymmetry, indicating a true underlying lesion and prompting an upgrade to BI-RADS 4. F and H are CC and MLO CEM images of contra lateral normal breast. Targeted ultrasound further supports this upgrade: Doppler imaging (I) demonstrates internal vascularity, while grayscale ultrasound (J) shows architectural distortion with posterior acoustic shadowing, both features concerning for malignancy. Histopathology (K) confirms the diagnosis of invasive lobular carcinoma. MLO: mediolateral oblique; CC: craniocaudal; BI-RADS: Breast Imaging Reporting and Data System; CEM: contrast-enhanced mammography

**Figure 3:**
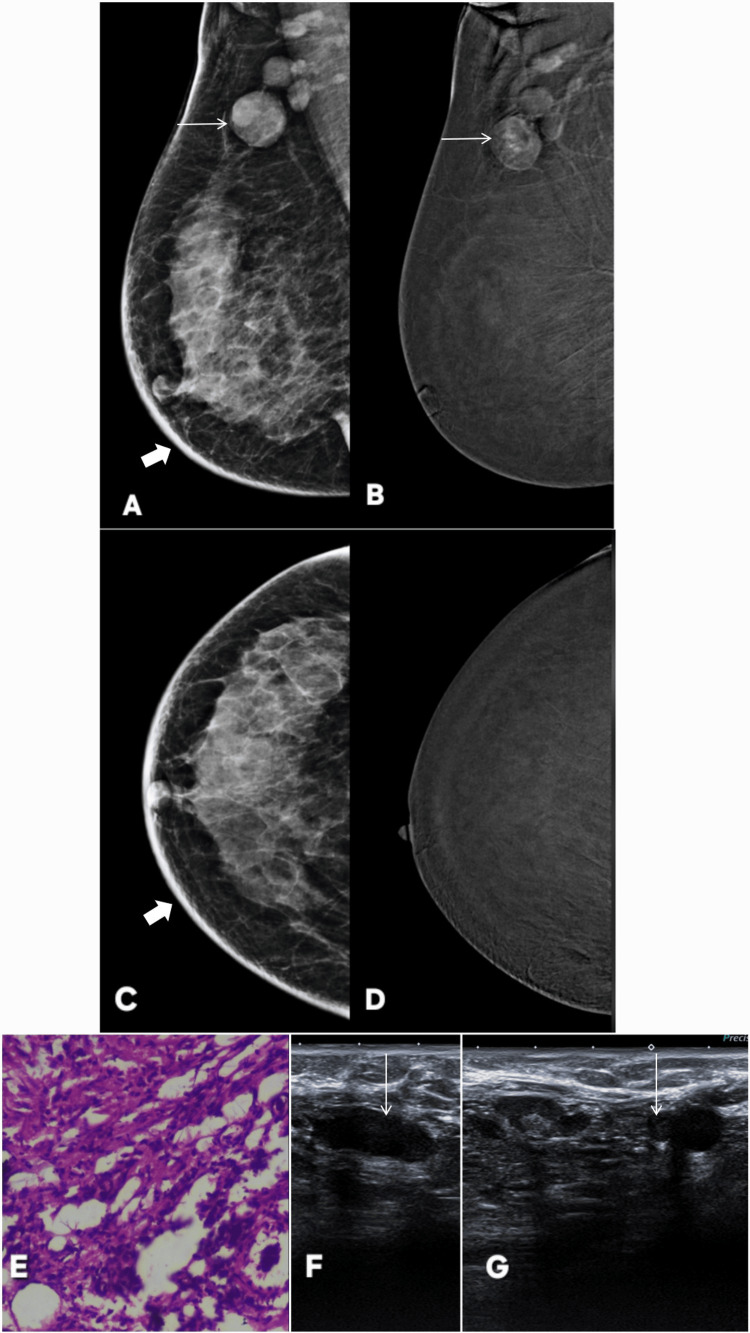
Contrast-enhanced mammography (CEM) in a patient with axillary tuberculosis demonstrating a true negative CEM finding. A 38-year-old female patient with positive family history and lump in right axilla. Panels A and C show digital mammography in mediolateral oblique and craniocaudal projections, respectively, while Panels B and D are the corresponding CEM images. Mammography reveals enlarged right axillary lymph nodes (thin white arrows), dense edematous breast parenchyma, and skin thickening (thick white arrows) (BI-RADS 0). No large focal mass or architectural distortion is identified. On CEM, there is no abnormal parenchymal enhancement, confirming the absence of any underlying breast lesion (BI-RADS 2). Ultrasound images (Panels F and G) demonstrate enlarged, hypoechoic right axillary lymph nodes. No focal breast parenchymal lesion was appreciable. Histopathology (Panel E) shows caseating necrosis, confirming tuberculous lymphadenitis. These findings establish that the breast parenchymal changes were reactive, secondary to axillary tuberculosis, with no intrinsic breast malignancy, thereby representing a true negative case on CEM. BI-RADS: Breast Imaging Reporting and Data System

**Figure 4:**
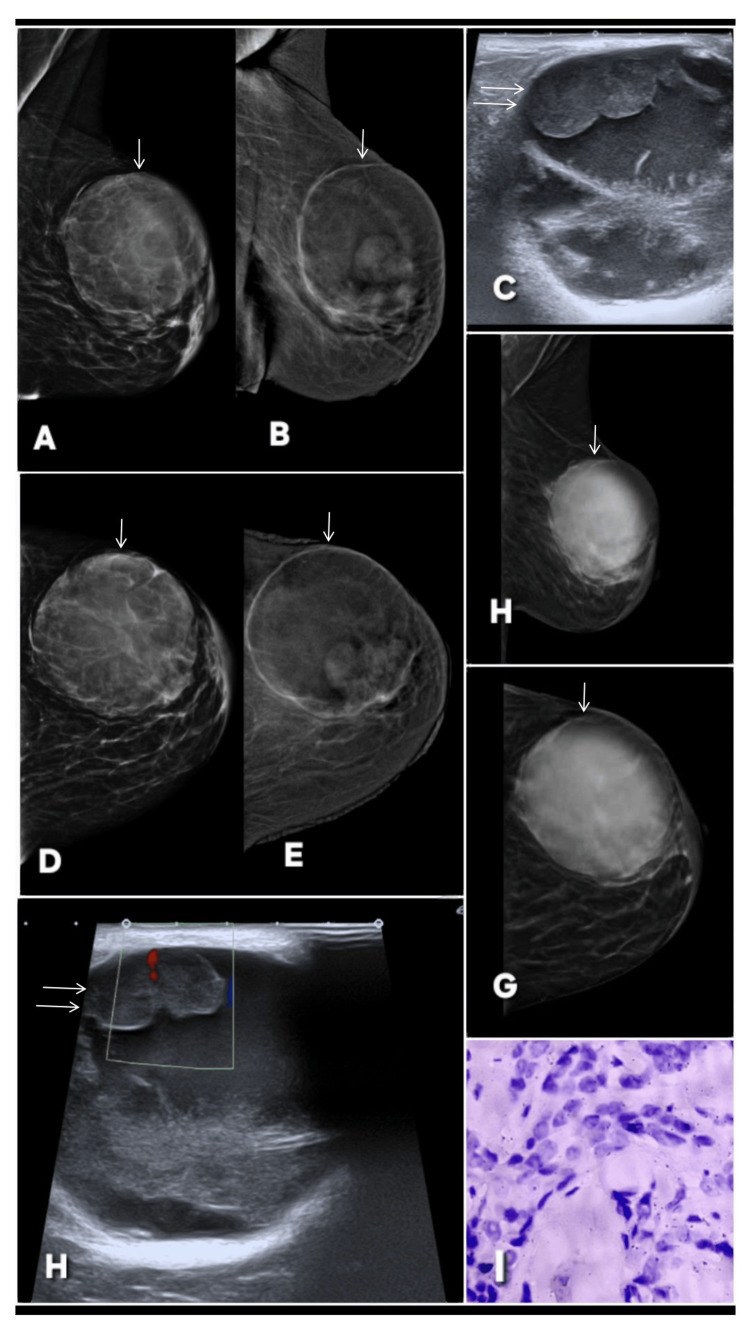
Correct upgrade of a complex cystic lesion on CEM in a patient with a rapidly growing lump in left breast. The lesion is marked by white arrows across all radiologic images. A 60-year-old female patient presented with lump in left breast rapidly increasing in size. Digital mammography images (A: MLO, D: CC) demonstrate a well-circumscribed cystic lesion with predominantly sharp margins, initially suggesting a benign etiology and categorized as BI-RADS 3. A perifocal lucent halo is present, and the lesion shows close abutment to the overlying skin, though no definite solid component is appreciable on conventional mammography. Contrast-enhanced mammography (B: MLO, E: CC), however, reveals asymmetric wall enhancement and a lobulated enhancing soft-tissue component arising from the inner wall of the cyst. These suspicious features prompted an upgrade to BI-RADS 4, highlighting the added value of CEM in characterizing complex cystic lesions. Gray scale ultrasound (C) confirms a complex cystic mass with a large internal cystic cavity containing low-level debris, thick internal septation, and a marginal lobulated solid component firmly attached to the cyst wall. Color Doppler imaging (H) further demonstrates internal vascularity within the solid portion, elevating concern for malignancy. Digital breast tomosynthesis (F: MLO, G: CC) delineates the cystic morphology but fails to demonstrate the intralesional solid component, underscoring the superiority of CEM in detecting enhancing malignant elements within complex cysts. Histopathology (I) confirms the imaging suspicion, showing features consistent with invasive ductal carcinoma. MLO: mediolateral oblique; CC: craniocaudal; BI-RADS: Breast Imaging Reporting and Data System; CEM: contrast-enhanced mammography

**Figure 5:**
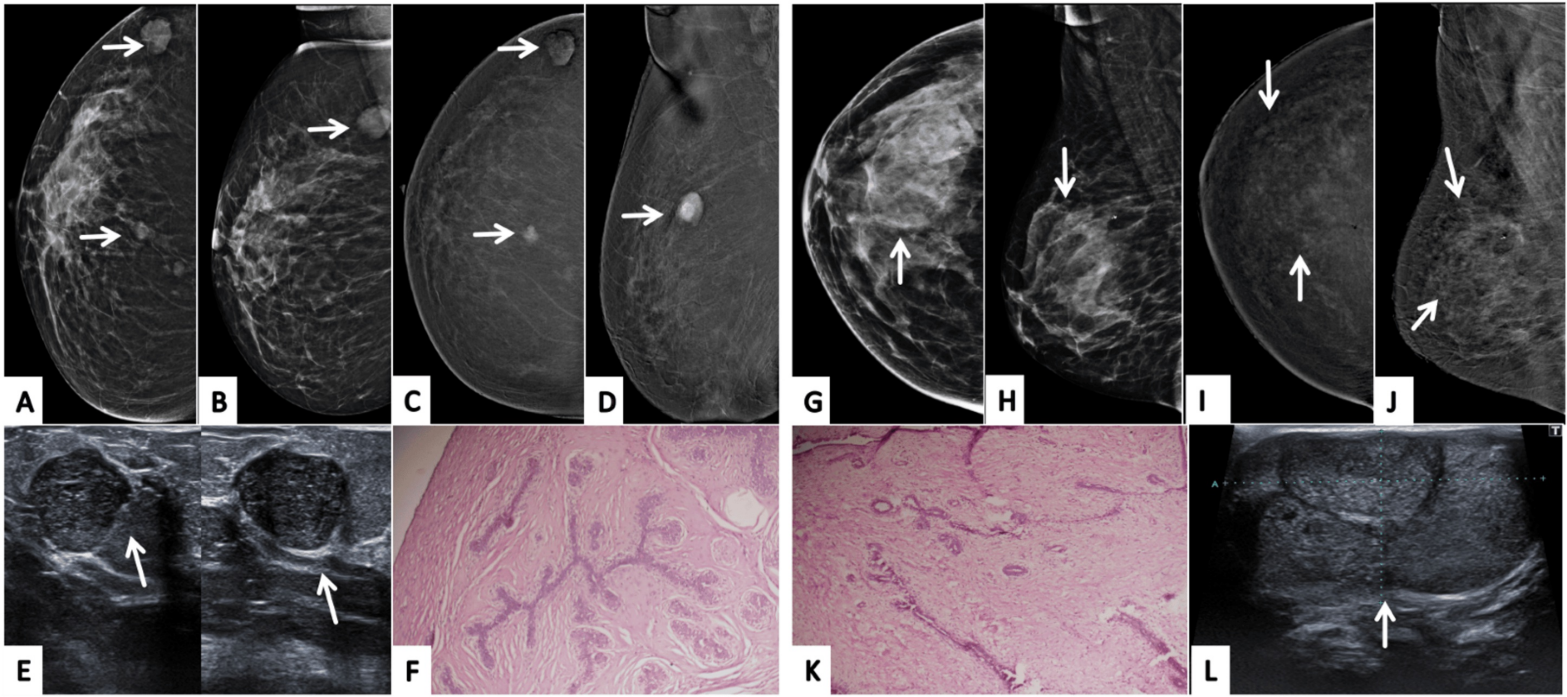
Variable enhancement patterns of fibroadenoma on contrast-enhanced mammography (CEM): examples of false-positive/incorrect upgrade and true-negative/correct downgrade lesions. Images are shown side by side for comparison, and lesions are annotated by white arrows. Patient α (Panels A–F): a 46-year-old female patient presented with lumpiness in right breast. Panels A and B show standard digital mammography of the right breast in mediolateral oblique (MLO) and craniocaudal (CC) projections, respectively, demonstrating two well-circumscribed, sharply marginated masses (BI-RADS 2). Corresponding CEM images (C and D) reveal contrast enhancement of these lesions upgrading these to BI-RADS 4. Ultrasound (E) demonstrates oval, well-defined, homogeneous hypoechoic lesions with features consistent with fibroadenoma. Histopathology (F) confirmed the diagnosis of fibroadenoma re-affirming the benignity of lesion. This represents a false-positive case on CEM, where benign fibroadenomas showed enhancement, emphasizing the need to rely on morphologic features over enhancement when the lesion is well circumscribed and non-infiltrative. Patient β (Panels G–L): a 45-year-old female patient presented with palpable lump in right breast. Panels G and H show standard digital mammography in MLO and CC views demonstrating a well-circumscribed lesion in the upper outer quadrant of the breast (BI-RADS 3). Corresponding CEM images (I and J) show no contrast enhancement of this lesion  correctly downgrading the lesion to BI-RADS 2. Ultrasound (L) demonstrates an oval, wider-than-tall, well-defined hypoechoic lesion. Histopathology (K) confirmed fibroadenoma. These images demonstrates that fibroadenomas may or may not enhance on CEM, reinforcing that enhancement alone is not specific for malignancy and should be interpreted in conjunction with morphology and other imaging features. MLO: mediolateral oblique; CC: craniocaudal; BI-RADS: Breast Imaging Reporting and Data System; CEM: contrast-enhanced mammography

**Figure 6:**
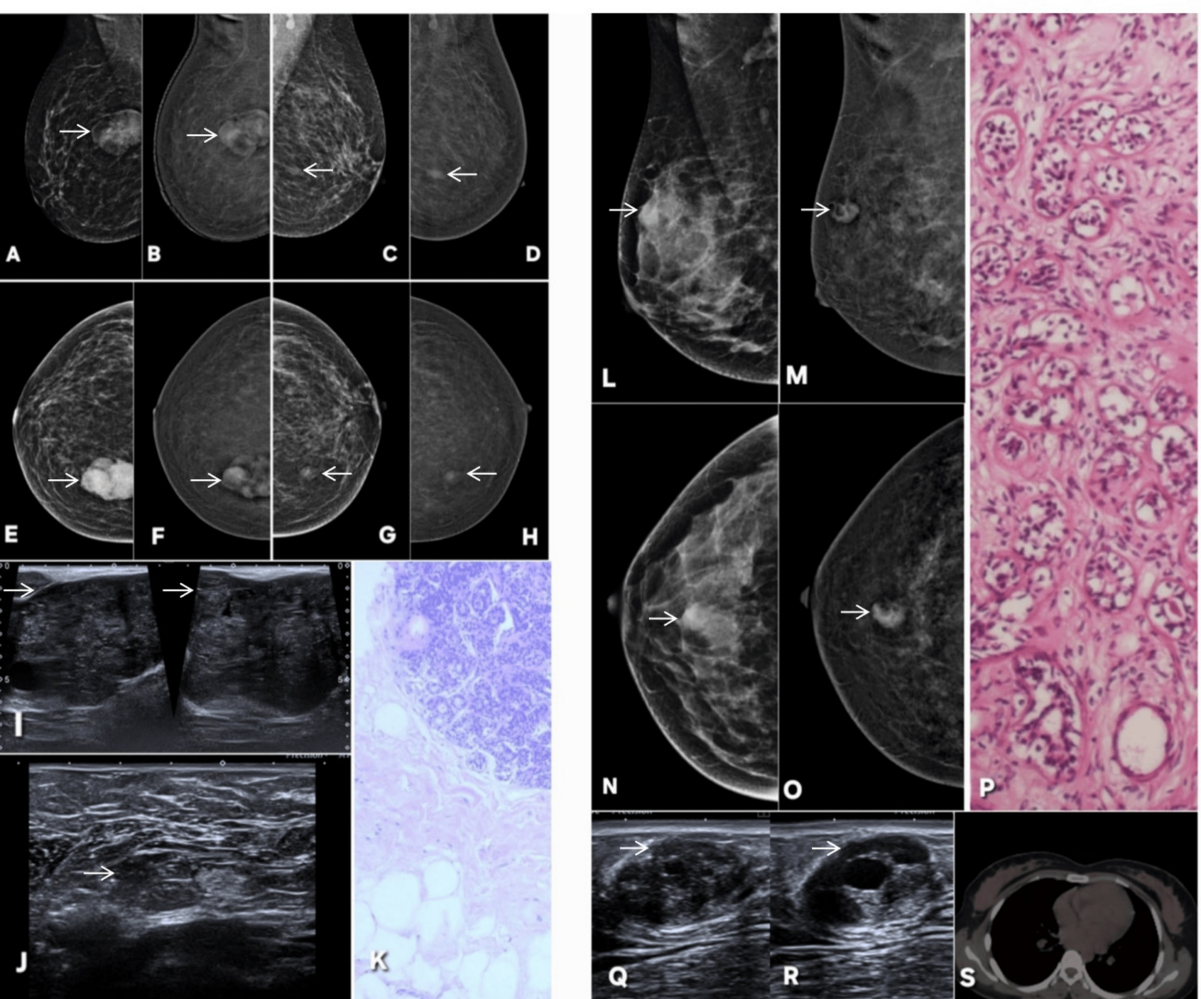
Examples of false-positive enhancement on contrast-enhanced mammography (CEM) with benign histopathological correlation. Lesion is annotated by white arrows in all radiological images. Patient γ (Panels A–K): a 40-year-old woman presented with breast lumps. Panels A and B show right breast MLO views on DM and CEM, respectively, while panels C and D show left breast MLO views. Panels E and F show right breast CC views on DM and CEM, respectively. Panels G and H depict respective left breast CC views. Bilateral lesions are noted on DM as well-circumscribed, wider-than-tall, smoothly marginated masses (BI-RADS 2). Right- and left-sided lesions are shown on ultrasound in panels I and J, respectively. However, CEM demonstrated enhancement within lesions upgrading them to BI-RADS 4. Histopathology (K) confirmed sclerosing adenosis. This represents a false-positive finding on CEM, where benign adenosis demonstrated enhancement, underscoring that in such well-defined, tissue-aligned, and non-infiltrative lesions, morphologic features should be prioritized over enhancement patterns to avoid overestimation of malignancy. Patient δ (Panels L–S): a 47-year-old woman, undergoing chemotherapy for non-Hodgkin lymphoma, developed a palpable lump during treatment, raising suspicion for disease progression. However, the imaging–pathology correlation confirmed benignity. Panels L and M show right breast MLO views on DM and CEM, respectively, while panels N and O represent CC projections. A well-circumscribed lesion is seen in the central upper quadrant on DM (BI-RADS 2). Again, on CEM it is seen demonstrating enhancement upgrading it to BI-RADS 4. Ultrasound (Q and R) shows a wider-than-tall, smooth-margined, benign-appearing lesion. Histopathology (P) confirmed proliferative breast disease without malignancy, and PET-CT (S) showed no metabolic uptake in the lesion. This highlights another false-positive CEM finding, emphasizing the importance of integrating morphologic and clinical context to prevent misinterpretation of enhancement as malignancy. MLO: mediolateral oblique; CC: craniocaudal; BI-RADS: Breast Imaging Reporting and Data System; DM: digital mammography

**Figure 7:**
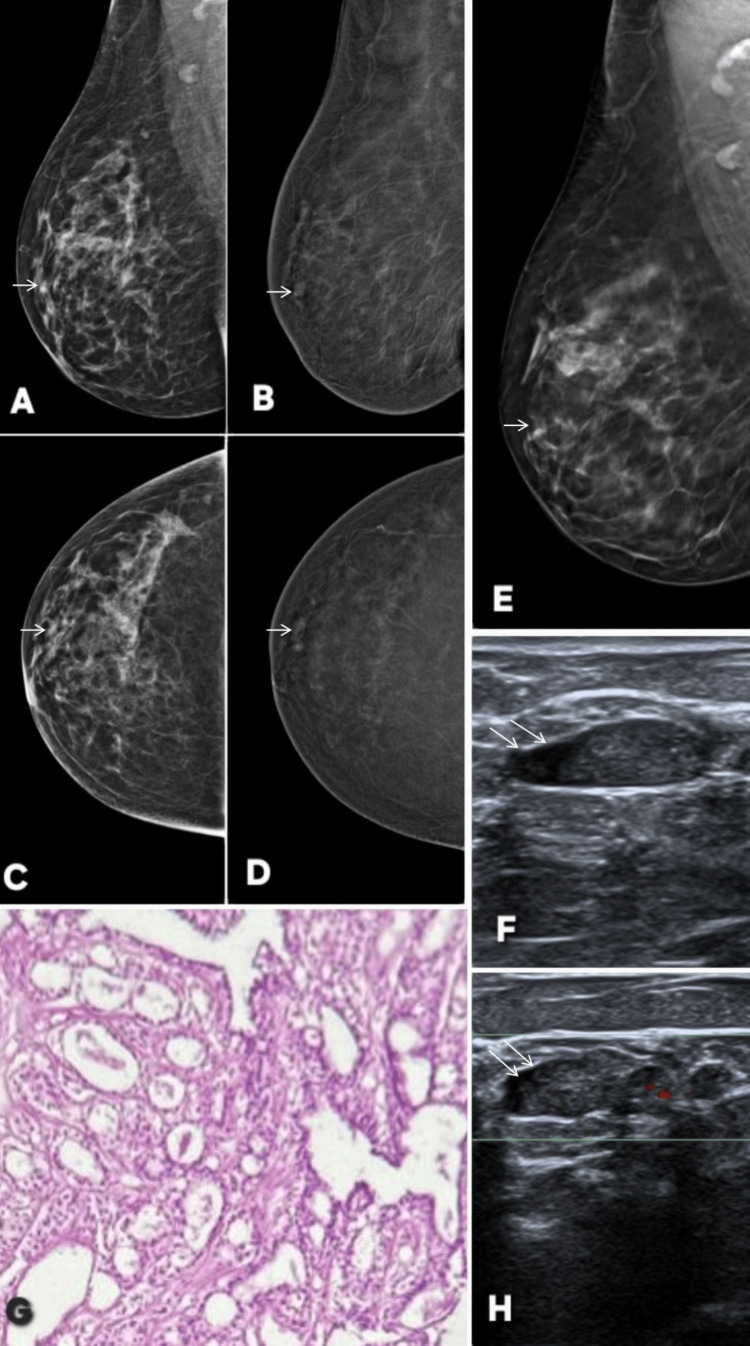
False-positive upgrade on CEM in a patient with nipple discharge. The lesion is annotated by white arrows in all radiological images. A 60-year-old female patient presented with unilateral nipple discharge and a positive family history of breast cancer. Digital mammography (A: MLO, C: CC) demonstrates a discrete, prominent linear duct in the upper outer quadrant. The background breast parenchyma is unremarkable, with no associated pleomorphic microcalcifications or architectural distortion. Based solely on mammography, the finding was categorized as BI-RADS 3, reflecting a probably benign dilated duct. Contrast-enhanced mammography (B: MLO, D: CC) reveals focal enhancement within the dilated duct, prompting an upgrade to BI-RADS 4 due to concern for an intraductal malignant process. Digital breast tomosynthesis (E) again delineates the dilated duct but does not provide additional characterization of the internal component. Ultrasound (F) further clarifies the nature of the lesion, showing a dilated duct harboring an intraluminal solid component, while color Doppler imaging (H) confirms internal vascularity within this intraductal tissue-features commonly associated with a suspicious or neoplastic process. Histopathology (G), however, demonstrates intraductal papilloma, confirming a benign etiology and classifying this case as a false-positive CEM upgrade. MLO: mediolateral oblique; CC: craniocaudal; BI-RADS: Breast Imaging Reporting and Data System; CEM: contrast-enhanced mammography

A series of collages demonstrates representative examples of correct CEM upgrades in Figures 1-4. Figures 5-7 demonstrate incorrect upgrades. 

**Figure 8:**
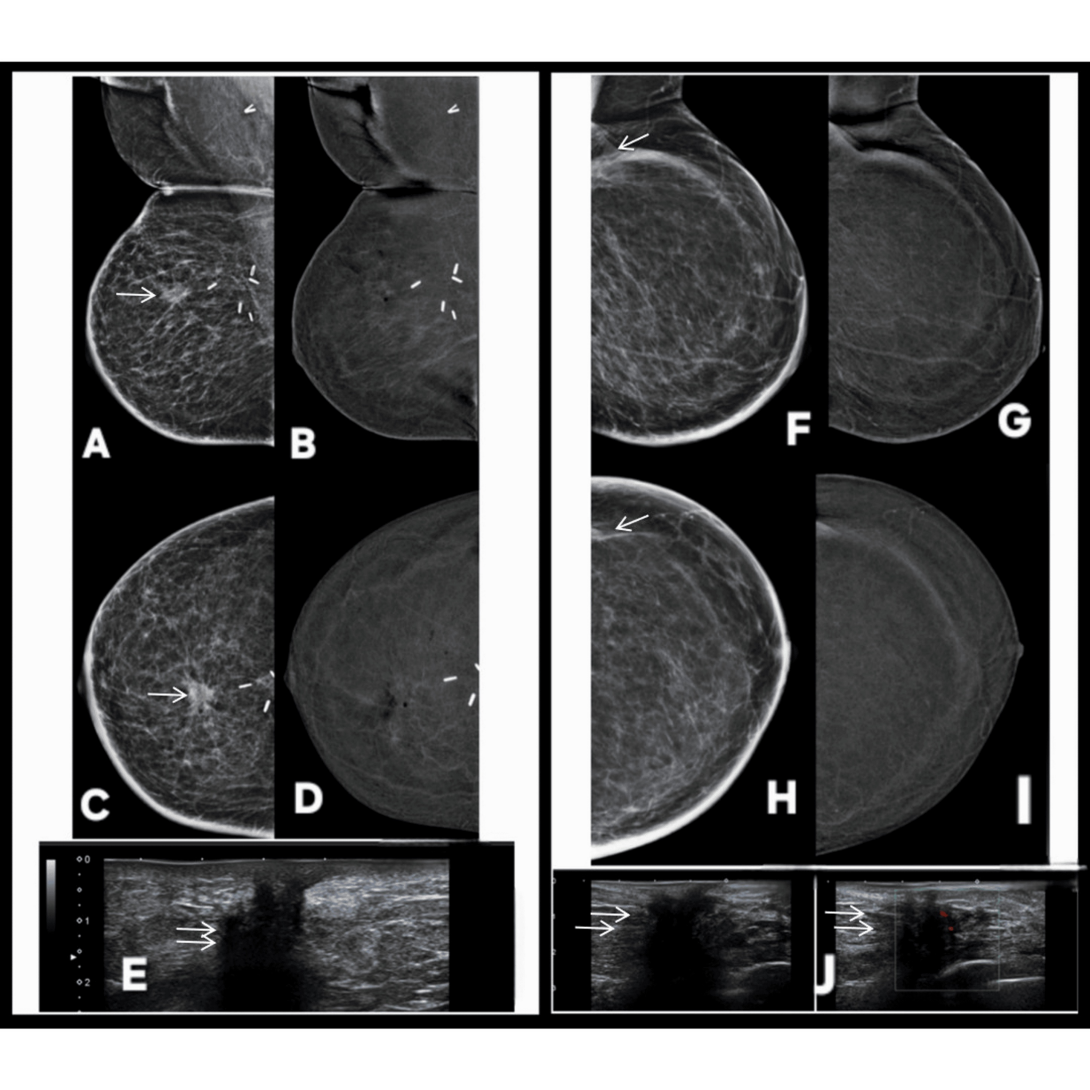
Contrast-enhanced mammography (CEM) as a problem-solving tool in post-surgical architectural distortion: two cases. Described abnormality is annotated by white arrows in all radiological images. Patient ε (Panels A–E): A 50-year-old woman, a three-year post–breast-conserving surgery case for invasive lobular carcinoma, presented for routine surveillance with no available prior imaging for comparison. Digital mammography (DM) in MLO and CC projections (A, C) demonstrates post-surgical clips and an ill-defined area of architectural distortion in the upper inner quadrant (white arrows). Given the oncologic history and absence of priors, the finding was categorized as BI-RADS 3. CEM (B, D) shows complete absence of contrast enhancement at the site of mammographic concern, effectively downgrading the abnormality to BI-RADS 2. Targeted grayscale ultrasound (E) reveals ill-defined posterior acoustic shadowing deep to surgical scar, initially appearing suspicious. However, due to non-enhancing CEM, the finding was reclassified as likely benign post surgical change. At six-month follow-up, stable sonographic appearance is seen, confirming benignity. Patient ζ (Panels F–J): a 55-year-old woman, six-month post–breast-conserving surgery case, was undergoing early routine follow-up. DM images (F, H) show ill-defined area of architectural distortion in the upper outer quadrant (white arrows), suspicious in appearance (BI-RADS 4). Grayscale ultrasound (J) shows a poorly marginated hypoechoic area without definable mass, likewise requiring additional evaluation. CEM (G, I), however, shows no enhancing mass or non-mass lesion, indicating absence of active pathological enhancement and supporting a benign postoperative etiology (BI-RADS 2). At six months, findings remained stable, corroborating the benign interpretation. In both cases, CEM provided critical diagnostic clarification when DM and ultrasound demonstrated architectural distortion with suspicious or indeterminate morphology. Lack of enhancement on CEM allowed confident downgrading preventing unnecessary biopsies. MLO: mediolateral oblique; CC: craniocaudal; BI-RADS: Breast Imaging Reporting and Data System

**Figure 9:**
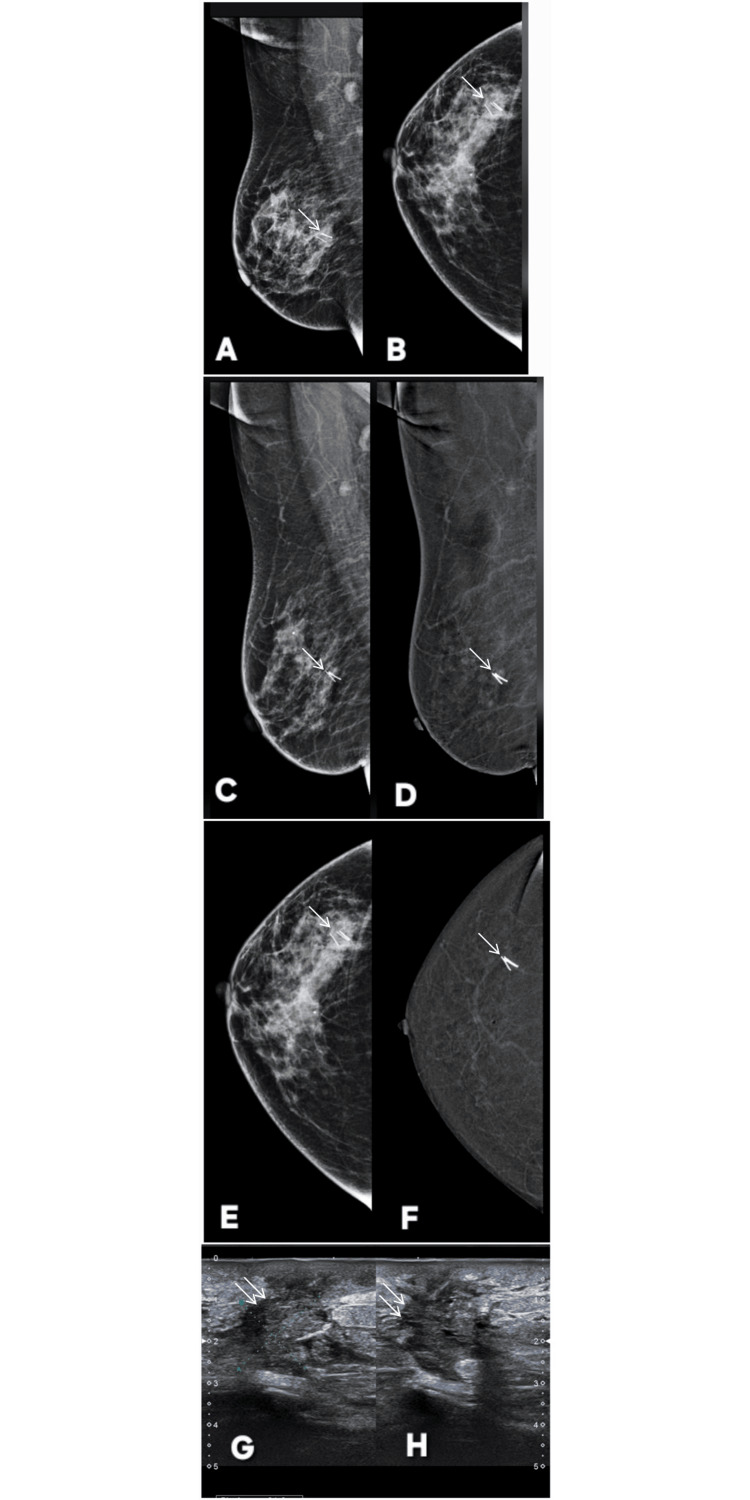
Assessment of post-chemotherapy response using digital mammography (DM), contrast-enhanced mammography (CEM), and ultrasound. The described abnormality is annotated by white arrows in all radiological images. The patient, a 41 years female, is a known case of invasive ductal carcinoma who underwent clip placement prior to neoadjuvant chemotherapy. Pre-treatment imaging (A, B): BI-RADS 6 digital mammography (MLO and CC) demonstrates metallic localization clips within the upper outer quadrant (white arrows). Due to asymmetrically increased background parenchymal density in this region, the primary lesion is poorly delineated, limiting confident assessment of the tumor margins on DM alone. Post-chemotherapy digital mammography (C, E): After eight cycles of neoadjuvant chemotherapy, DM in MLO and CC projections shows marked interval reduction (“melting”) of the previously noted density, particularly evident on the MLO view. Only minimal surrounding architectural distortion persists, consistent with treatment response (BI-RADS 3). Post-chemotherapy CEM (D, F): CEM performed at the same time demonstrates complete absence of contrast enhancement at the site of the tumor bed and clips. The lack of enhancement strongly suggests no residual viable disease, reinforcing the subtle findings seen on DM and illustrating the ability of CEM to confidently exclude residual enhancing tumor post-therapy (BI-RADS 2). Targeted ultrasound (G, H): Ultrasound reveals post-treatment architectural distortion with posterior shadowing and the metallic clips in situ (white arrows). No definable mass or focal lesion is identified, further supporting the absence of residual tumor and correlating directly with CEM findings. The combined findings across modalities demonstrate a robust therapeutic response with no residual enhancing disease on CEM, confirming its value as a reliable tool for post-chemotherapy assessment, especially when DM and ultrasound show treatment-related distortion without a discrete mass. MLO: mediolateral oblique; CC: craniocaudal; BI-RADS: Breast Imaging Reporting and Data System

**Figure 10:**
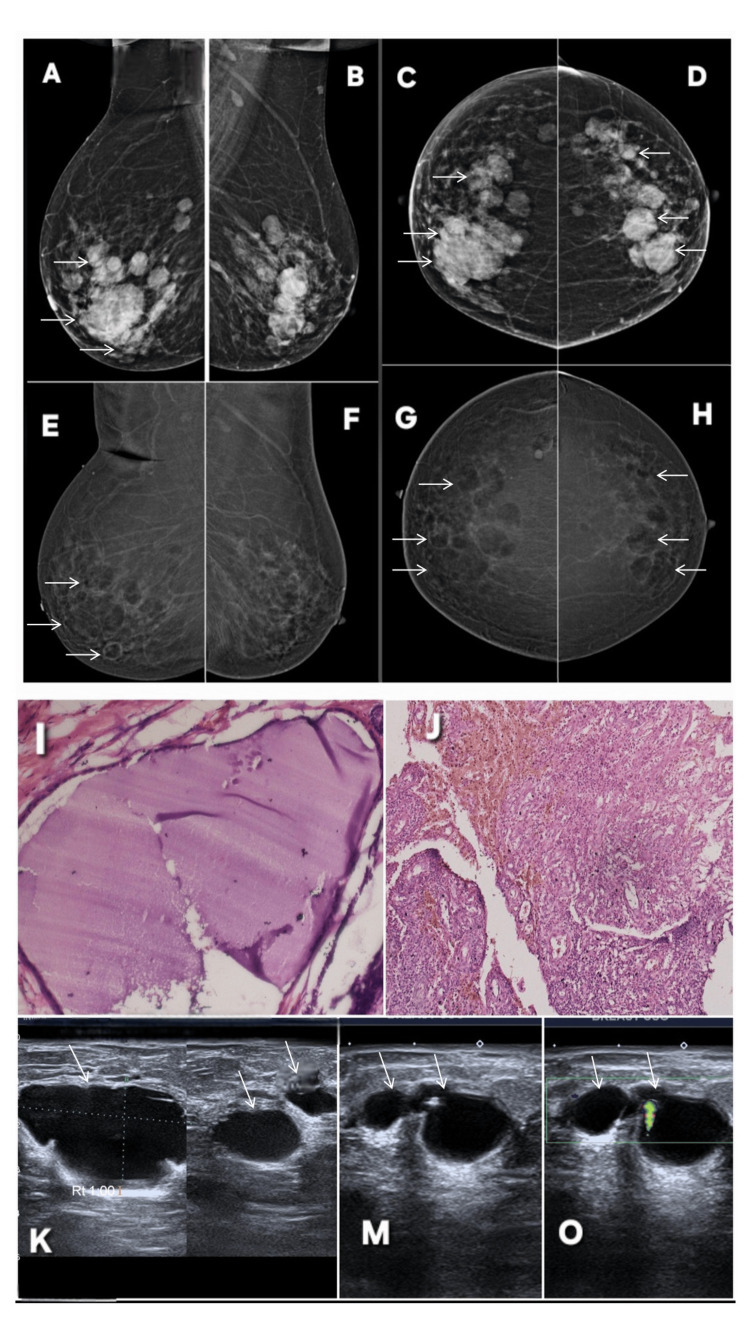
True negative case (multiple breast cysts with one infected cyst). Lesion is annotated by white arrows in all radiological images. Digital mammography of both breasts of a 43-year-old female patient, (A, B) MLO views and (C, D) CC views, demonstrate multiple, well-circumscribed, rounded to oval masses of varying sizes scattered throughout both breasts (white arrows). These lesions show smooth margins and no associated architectural distortion or suspicious microcalcifications (BI-RADS 3). CEM ((E, F: MLO; G, H: CC) reveal multiple non-enhancing, well-defined cystic lesions bilaterally (white arrows), showing smooth peripheral margins without internal enhancing septations or solid components, confirming their benign cystic nature (BI-RADS 2). However, in the right breast, one cyst demonstrates focal, irregular peripheral wall enhancement (double white arrows in E, G), suggestive of inflammatory change or early infection. No suspicious parenchymal or ductal enhancement is observed elsewhere. Gray scale ultrasound evaluation of the right breast (K–M) demonstrates anechoic to minimally complex cystic lesions with thin walls (white arrows). The cysts show posterior acoustic enhancement and smooth internal margins, consistent with simple cysts. One cyst exhibits mild wall thickening and internal echoes, raising suspicion for infection or inflammation. On color Doppler (M), there is peripheral vascularity along the cyst wall, confirming active inflammatory process. A comet-tail artifact is noted adjacent to a tiny calcification within another cyst, confirming benign microcalcification. Histopathology findings (I, J) confirm the imaging impressions. Section from a simple cyst (I) reveals a cystic cavity lined by benign cuboidal to flattened epithelium with no evidence of atypia or malignancy. Section from the right breast cyst wall (J) shows dense inflammatory infiltrate with neutrophilic exudate and granulation tissue, consistent with mastitis secondary to cyst infection. MLO: mediolateral oblique; CC: craniocaudal; BI-RADS: Breast Imaging Reporting and Data System; CEM: contrast-enhanced mammography

**Figure 11:**
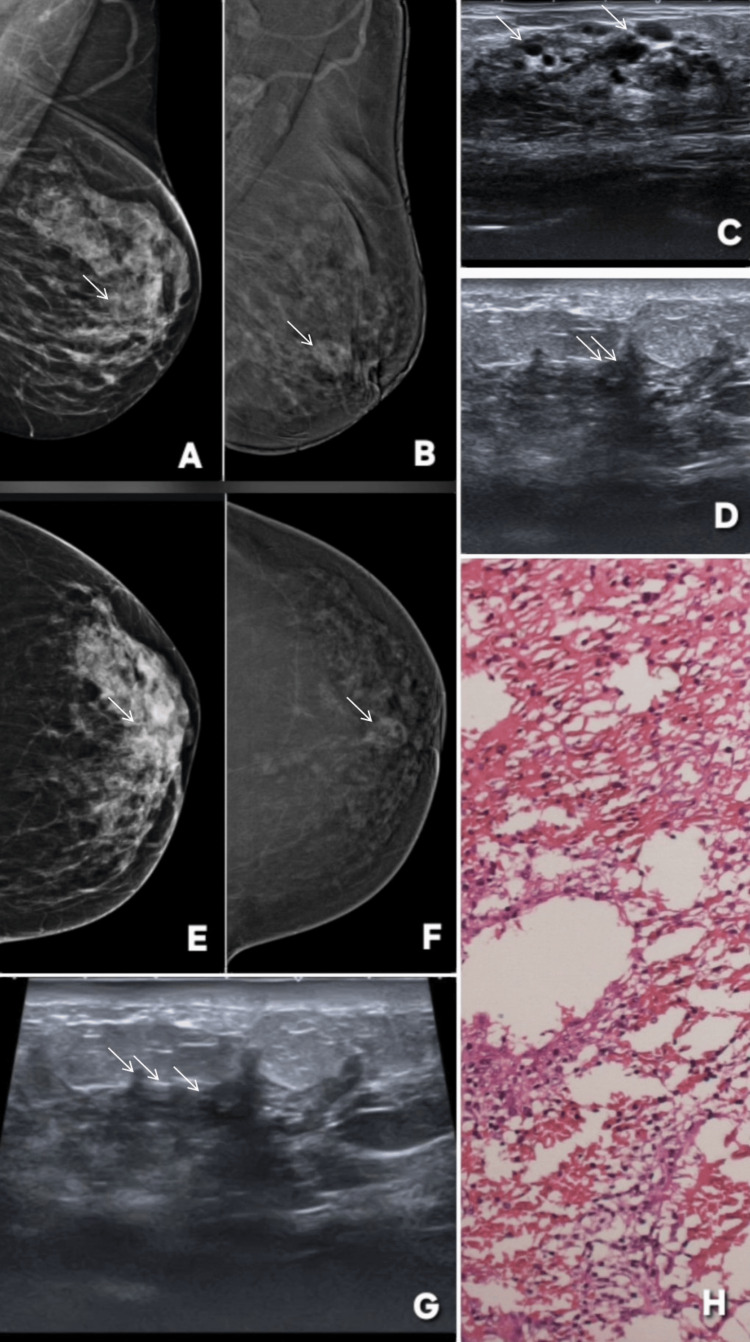
Correct downgrade on CEM in mastitis/ductitis. The relevant abnormality is highlighted with white arrows in all radiological images. A 43-year-old female patient presented with breast pain, focal erythema, and tenderness. Digital mammography (A: MLO; E: CC) shows a prominent, thick-walled duct in the affected quadrant, with adjacent asymmetric parenchymal density. No suspicious microcalcifications or mass-forming lesion are seen. Based on these findings, the abnormality was initially categorized as BI-RADS 3 (probably benign), with duct ectasia or early inflammatory change as differential considerations. Contrast-enhanced mammography (B: MLO; F: CC) further refines the assessment by demonstrating mild, smooth, linear duct-wall enhancement without a focal enhancing mass or nodular component. No segmental, clumped, or mass-like enhancement is present. The enhancement pattern reflects reactive inflammatory hyperemia, consistent with benign ductitis, rather than malignancy. CEM therefore correctly downgrades the finding from BI-RADS 3 to BI-RADS 2 (benign). Targeted ultrasound provides additional confirmation. Panel C shows a dilated duct with internal mobile echogenic debris, compatible with inscipissated secretions or inflammatory material. Panel D reveals an ill-defined hypoechoic area with surrounding edema, typical of acute mastitis. Ultrasound (G) demonstrates heterogeneous inflamed parenchyma, loss of normal tissue planes, and increased vascularity (not shown), further supporting an inflammatory rather than neoplastic process. Histopathology (H) confirms the diagnosis of mastitis/ductitis, consistent with the imaging features. MLO: mediolateral oblique; CC: craniocaudal; BI-RADS: Breast Imaging Reporting and Data System; CEM: contrast-enhanced mammography

**Figure 12:**
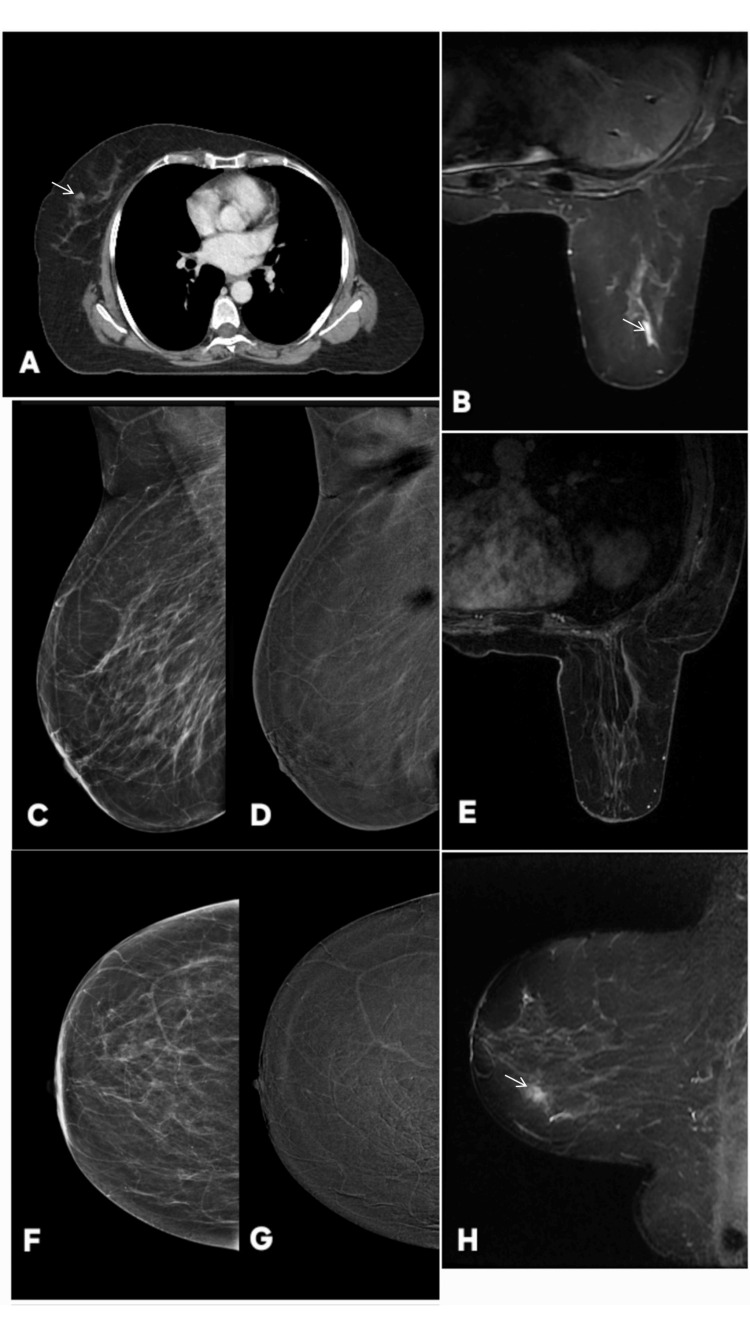
Contrast-enhanced mammography (CEM) correctly downgrading a suspicious focal enhancement in a 60-year-old surveillance female patient with treated left-breast invasive lobular carcinoma. The abnormality is annotated by white arrows in all images. A: Routine follow-up contrast-enhanced CT (axial) at level of right breast shows ill-defined focal area of parenchymal enhancement (white arrow). Although this could represent a summation artifact, an evolving focal abnormality could not be excluded, prompting further evaluation with breast MRI. Unfortunately no previous CT is available for comparison. B & H: Contrast-enhanced T1-weighted MRI of the right breast (axial in B; sagittal in H) demonstrates a linear enhancing focus in the same region, raising concern for a potentially enhancing duct or subtle non-mass enhancement. The configuration remained suspicious, necessitating correlation with mammography and CEM (BI-RADS 4). E: Corresponding T2-weighted axial MRI shows no discrete abnormality at the site of concern, resulting in discordant multiparametric MRI features and maintaining diagnostic uncertainty. C & F: Digital mammography (MLO and CC) demonstrates no focal mass, architectural distortion, or asymmetry at the site marked on CT/MRI. Parenchymal pattern remains stable compared with prior examinations (BI-RADS 2). D & G: CEM (MLO and CC) shows no areas of abnormal contrast uptake, excluding an enhancing mass or non-mass lesion. No focal parenchymal enhancement is seen on CEM subtraction images, and breast vascularity is normal (BI-RADS 2). Targeted ultrasound (not shown) also demonstrated no correlating abnormality. Despite suspicious focal enhancement on CT and equivocal linear enhancement on MRI, CEM demonstrated complete absence of enhancing parenchymal abnormality, allowing confident downgrading of the finding. Ultrasound after six months revealed normal breast. This underscores the value of CEM as a highly effective problem-solving tool, particularly when MRI findings are subtle, discordant, or inconclusive, and highlights its usefulness in surveillance imaging of patients with history of lobular carcinoma. MLO: mediolateral oblique; CC: craniocaudal; BI-RADS: Breast Imaging Reporting and Data System

**Figure 13:**
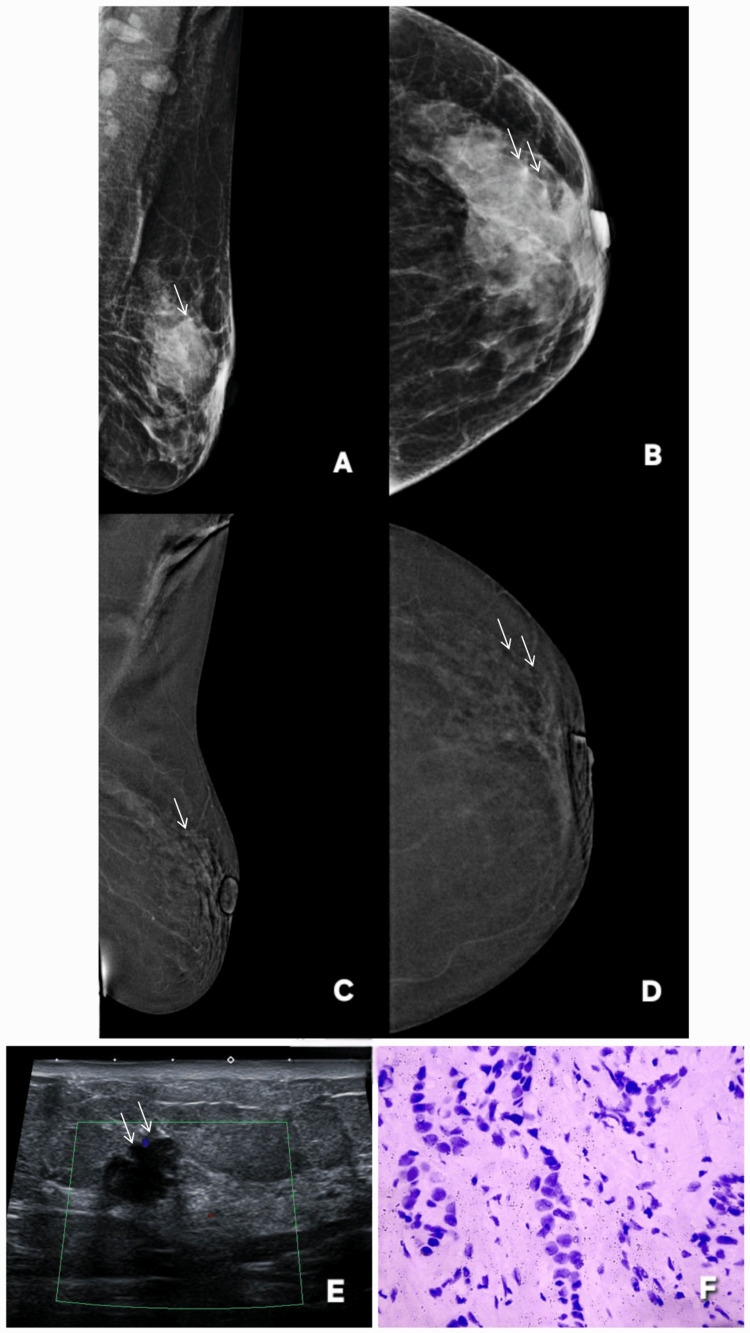
Multimodality imaging of invasive ductal carcinoma of the breast of a 55 years female, showing corresponding findings on mammography, contrast-enhanced mammography (CEM), ultrasound, and histopathology. The lesion is indicated by white arrows. (A, B) Digital Mammography (Mediolateral Oblique and Craniocaudal Views): Images show an irregular, high-density mass with indistinct margins located in the upper outer quadrant of the breast. The lesion causes surrounding architectural distortion, consistent with a highly suspicious malignancy (BI-RADS 5). (C, D) Contrast-Enhanced Mammography (CEM): Recombined CEM images demonstrate a non-enhancing irregular mass reflecting poor vascularity. No additional enhancing satellite or multifocal lesions are visualized. (E) Ultrasound: Targeted ultrasound reveals an irregular, hypoechoic mass with angular and spiculated borders, posterior acoustic shadowing, and taller-than-wide orientation. These sonographic features are characteristic of a malignant lesion. (F) Histopathology (H&E stain): Microscopic examination confirms invasive ductal carcinoma, not otherwise specified (NOS). Tumor cells are arranged in cords and nests infiltrating fibrous stroma, exhibiting pleomorphic, hyperchromatic nuclei with prominent nucleoli and increased mitotic activity, consistent with a high-grade invasive carcinoma.

Correct downgrades in CEM are shown in Figures 8-12. A false-negative case with a non-enhancing carcinoma is shown in Figure 13.

Concordant findings between DM and CEM are shown in Figures 14-24.

**Figure 14:**
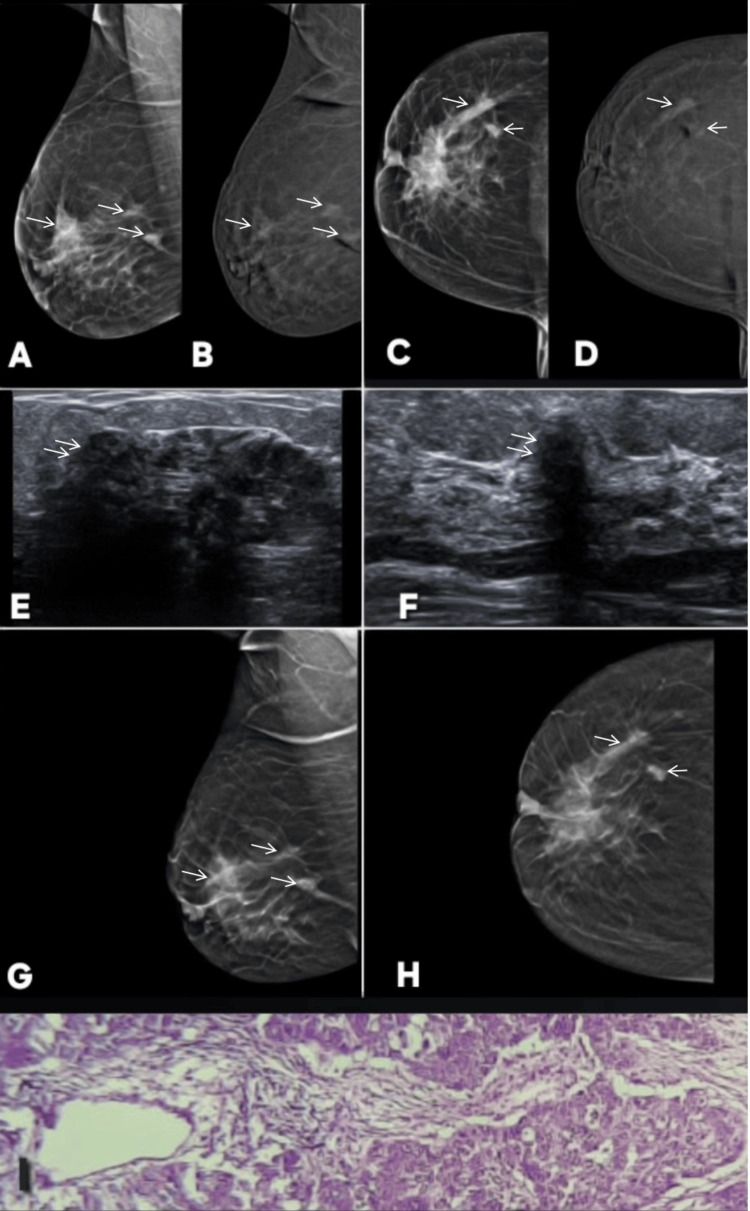
Concordant malignant findings on digital mammography (DM) and contrast-enhanced mammography (CEM). Lesions are marked with white arrows in all radiological images. A 50-year-old female patient presented with palpable lump in right breast. DM (A: MLO; C: CC) demonstrates two abnormal tubular opacities in the central and upper outer quadrants, corresponding to dilated ducts containing irregular soft-tissue components. These intraductal densities appear asymmetric and non-parallel, raising suspicion for a malignant process within the ductal system (BI-RADS 4). The corresponding CEM images (B: MLO; D: CC) show focal nodular and linear enhancement confined within these ducts, mirroring the DM findings. The enhancement pattern is heterogeneous and segmental, consistent with intraductal spread or early invasion. These features confirm the DM suspicion, demonstrating full concordance between DM and CEM in identifying malignant ductal pathology (BI-RADS 4). Targeted gray scale ultrasound (E, F) offers further characterization. Both images reveal dilated ducts containing irregular hypoechoic soft-tissue components. The intraductal tissue shows posterior acoustic shadowing and irregular extension through the ductal wall, findings highly suggestive of invasive ductal carcinoma with a ductal origin or spread. Digital breast tomosynthesis (G: MLO; H: CC) delineates the course of the ducts and again demonstrates the intraluminal soft-tissue components, supporting the mammographic and sonographic impressions by showing their true three-dimensional extent and ductal continuity. Histopathology (I) confirms invasive ductal carcinoma, correlating with the multimodality imaging appearances. MLO: mediolateral oblique; CC: craniocaudal; BI-RADS: Breast Imaging Reporting and Data System

**Figure 15:**
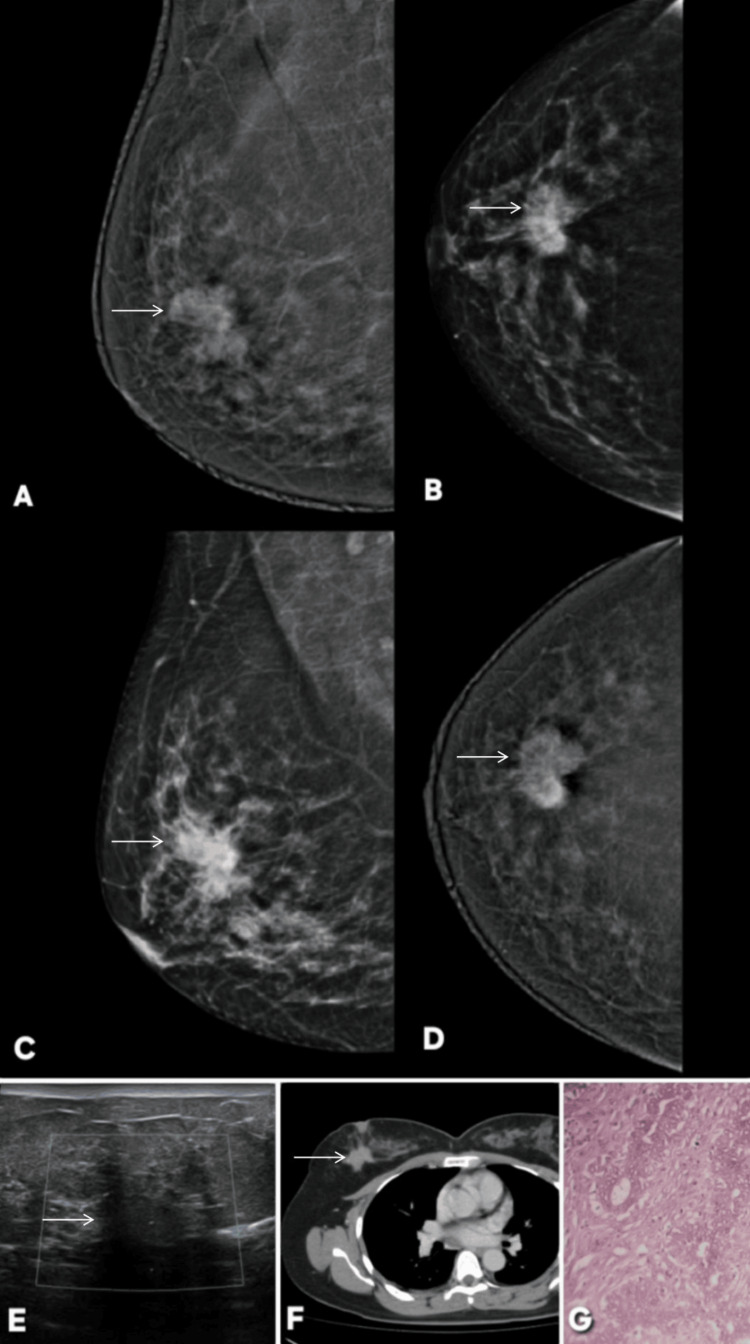
Concordant malignant findings on digital mammography (DM) and contrast-enhanced mammography (CEM). The lesion is annotated with white arrows in all radiological images. CEM of a 57-year-old female patient with right breast lump (A: MLO) demonstrates a spiculated, irregular enhancing mass in the upper outer quadrant of the breast. The lesion shows heterogeneous internal enhancement with radiating margins, consistent with a suspicious high-grade abnormality. The corresponding DM images (B: MLO; C: CC) reveal a dense, irregular mass with spicules extending into the surrounding parenchyma, characteristic of a BI-RADS 4C lesion. The lesion’s architectural distortion and radiating margins are clearly depicted, indicating a high suspicion for invasive malignancy. The CEM CC view (D) reproduces the same morphology with avid contrast uptake, confirming the presence of a vascular, infiltrative lesion. The enhancement pattern correlates precisely with the mammographic abnormality, demonstrating full concordance between modalities (BI-RADS 4c). Color Doppler ultrasound (E) shows intralesional vascularity within the solid component, further supporting the impression of an aggressive invasive tumor. The contrast-enhanced CT chest axial image (F) at the level of the main pulmonary vein demonstrates a focally enhancing mass in the upper outer quadrant, corresponding in location and morphology to the DM and CEM findings, indicating the lesion’s visibility across cross-sectional imaging. Histopathology (G) confirms invasive ductal carcinoma, matching the imaging characteristics. MLO: mediolateral oblique; CC: craniocaudal; BI-RADS: Breast Imaging Reporting and Data System; CEM: contrast-enhanced mammography

**Figure 16:**
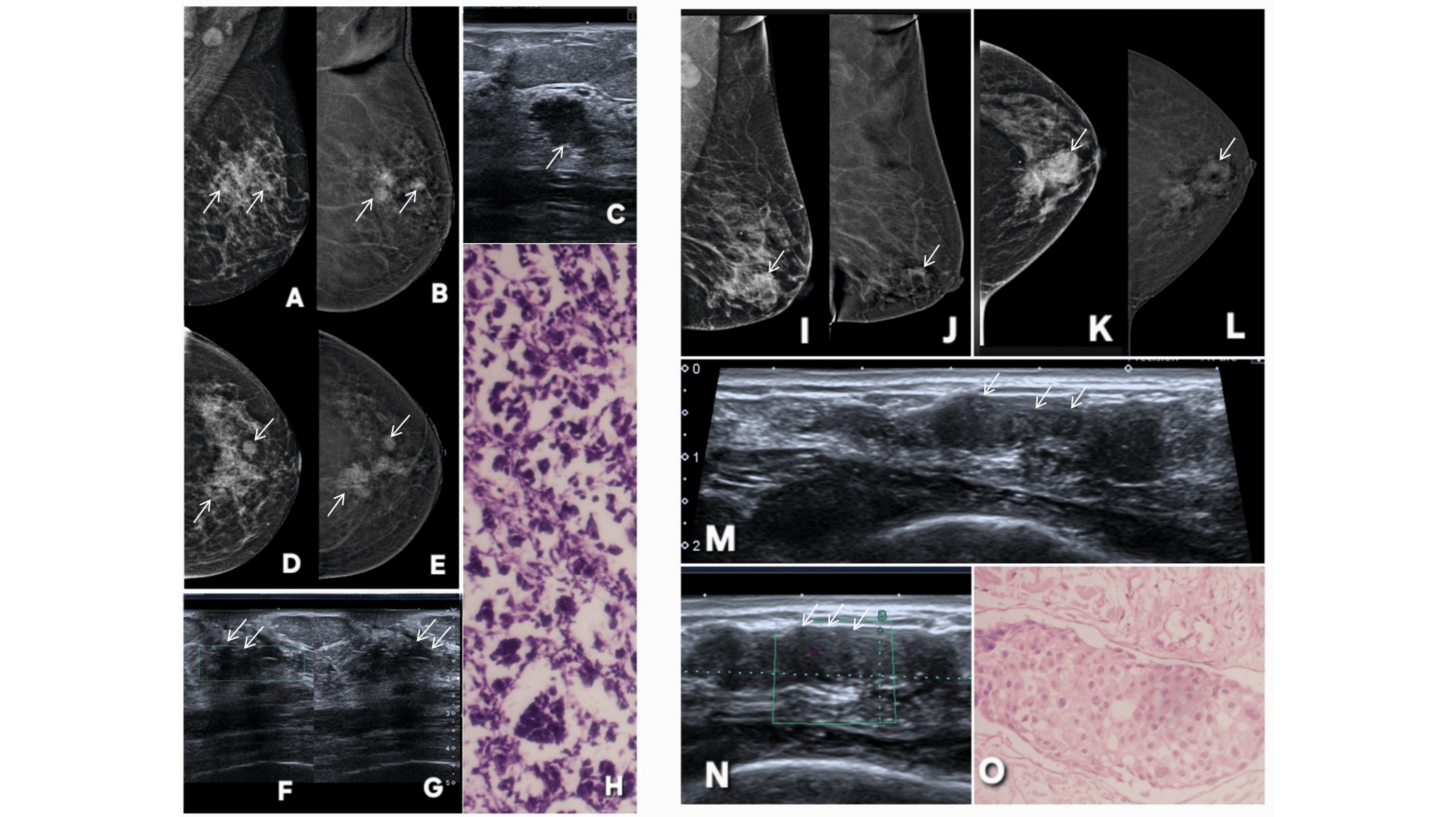
Representative cases demonstrating correlation between contrast-enhanced mammography (CEM) and histopathology findings. Described abnormality is annotated by white arrows in all radiological images. Patient κ (Panels A–H): A 48-year-old female patient presented with palpable lumps and pain. Panels A and D show standard digital mammography (DM) in the mediolateral oblique (MLO) and craniocaudal (CC) views, respectively. Panels B and E are the corresponding CEM images. Two lesions are identified in the lower inner quadrant of the breast: a branching enhancing lesion, corresponding to invasive mammary carcinoma, and a rounded lesion representing a metastatic intramammary lymph node (BI-RADS 4c). Ultrasound correlation is shown in Panels F and G, depicting the malignant lesion as an irregular, vascular mass on gray scale and color Doppler imaging. The metastatic intramammary lymph node is shown in Panel C. Histopathological examination (H) confirmed invasive mammary carcinoma with lymph node metastasis. These findings demonstrate excellent concordance between CEM enhancement characteristics and histopathological diagnosis. Patient λ (Panels I–O): A 54-year-old female patient presented with newly developed palpable lump. Panels I and K show DM in MLO and CC projections, while panels J and L are the corresponding CEM images. CEM demonstrates a ductal lesion with segmental enhancement expanding the duct, suspicious for a malignancy (BI-RADS 4c). Notably, associated microcalcifications remain non-enhancing. Ultrasound correlation (Panels M and N) demonstrates the corresponding lesion with internal vascularity on color Doppler. Histopathology (O) confirmed ductal carcinoma in situ arising within invasive ductal carcinoma. This case illustrates CEM–histopathology concordance for ductal malignancy while emphasizing that malignant microcalcifications may remain non-enhancing on CEM. MLO: mediolateral oblique; CC: craniocaudal; BI-RADS: Breast Imaging Reporting and Data System

**Figure 17:**
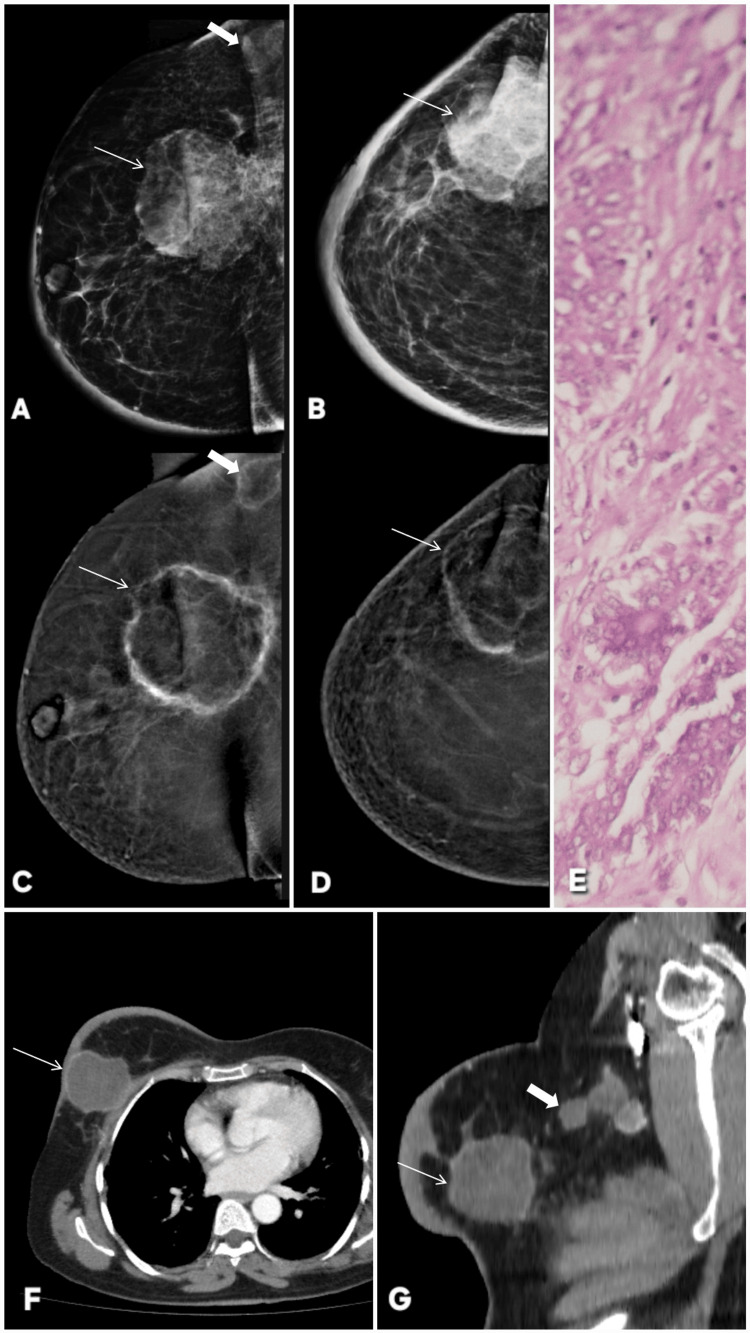
Imaging and histopathological findings in a 65-yearpold female patient with invasive ductal carcinoma. Lesion is annotated by white arrows in all radiological images. (A, B) Digital mammography showing a fairly well-circumscribed, high-density mass with irregular margins (thin white arrows) in the upper outer quadrant of the left breast, suggestive of a suspicious lesion with axillary lymphadenopathy (thick white arrows) (BI-RADS 5). (C, D) Contrast-enhanced mammography demonstrating avid peripheral enhancement with a central non-enhancing necrotic area (arrows), better delineating lesion margins and internal architecture compared with conventional mammography (BI-RADS 5). (E) Histopathological section (hematoxylin and eosin stain, ×20) showing malignant ductal epithelial cells arranged in irregular nests and cords within a desmoplastic stroma, consistent with invasive ductal carcinoma. (F, G) Contrast-enhanced CT images revealing a heterogeneously enhancing mass with central necrosis in the left breast (arrows) and ipsilateral axillary lymphadenopathy (double arrows), corresponding to the primary lesion and nodal metastasis, respectively. BI-RADS: Breast Imaging Reporting and Data System

**Figure 18:**
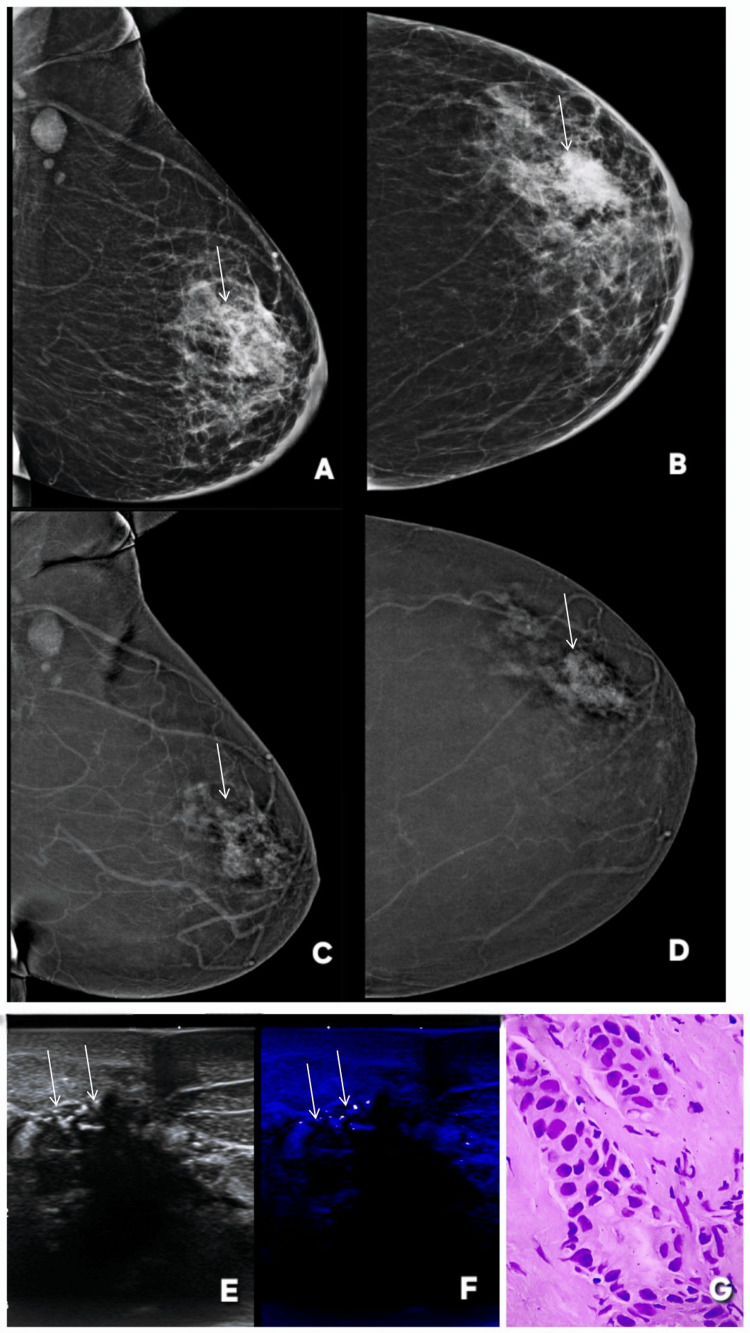
Multimodality imaging of invasive ductal carcinoma of the breast demonstrating mammographic, contrast-enhanced mammographic, ultrasonographic, and histopathologic correlation. Lesion is annotated by white arrows in all radiological images. A 63-year-old female patient presented with lump in left breast. (A and B) Digital mammography (mediolateral oblique and craniocaudal views): An irregular, high-density mass with spiculated margins and associated pleomorphic microcalcifications (arrow) is seen in the upper outer quadrant of the breast. There is associated architectural distortion, highly suspicious for malignancy (BI-RADS 5). (C and D) Contrast-enhanced mammography (CEM): Recombined CEM images show an irregular enhancing mass with heterogeneous internal enhancement and spiculated margins, corresponding to the mammographic lesion (BI-RADS 5). Notably, the associated microcalcifications demonstrate enhancement, consistent with enhancing malignant microcalcifications, a recognized feature of invasive ductal carcinoma. No additional enhancing foci or multifocal lesions are identified. (E) Ultrasound: Targeted ultrasound of the corresponding region demonstrates an irregular, hypoechoic mass with angular and spiculated margins, posterior acoustic shadowing, and non-parallel (taller-than-wide) orientation, consistent with malignancy. (F) Micropure ultrasound Image: Micropure imaging accentuates the presence of fine clustered microcalcifications within the mass (arrows), aiding better visualization of subtle calcific foci which are less conspicuous on conventional grayscale imaging. (G) Immunohistochemistry: Histopathological evaluation confirms invasive ductal carcinoma, not otherwise specified. Immunohistochemistry for progesterone receptor (PR) shows negative nuclear staining in tumor cells, indicating PR-negative status. BI-RADS: Breast Imaging Reporting and Data System

**Figure 19:**
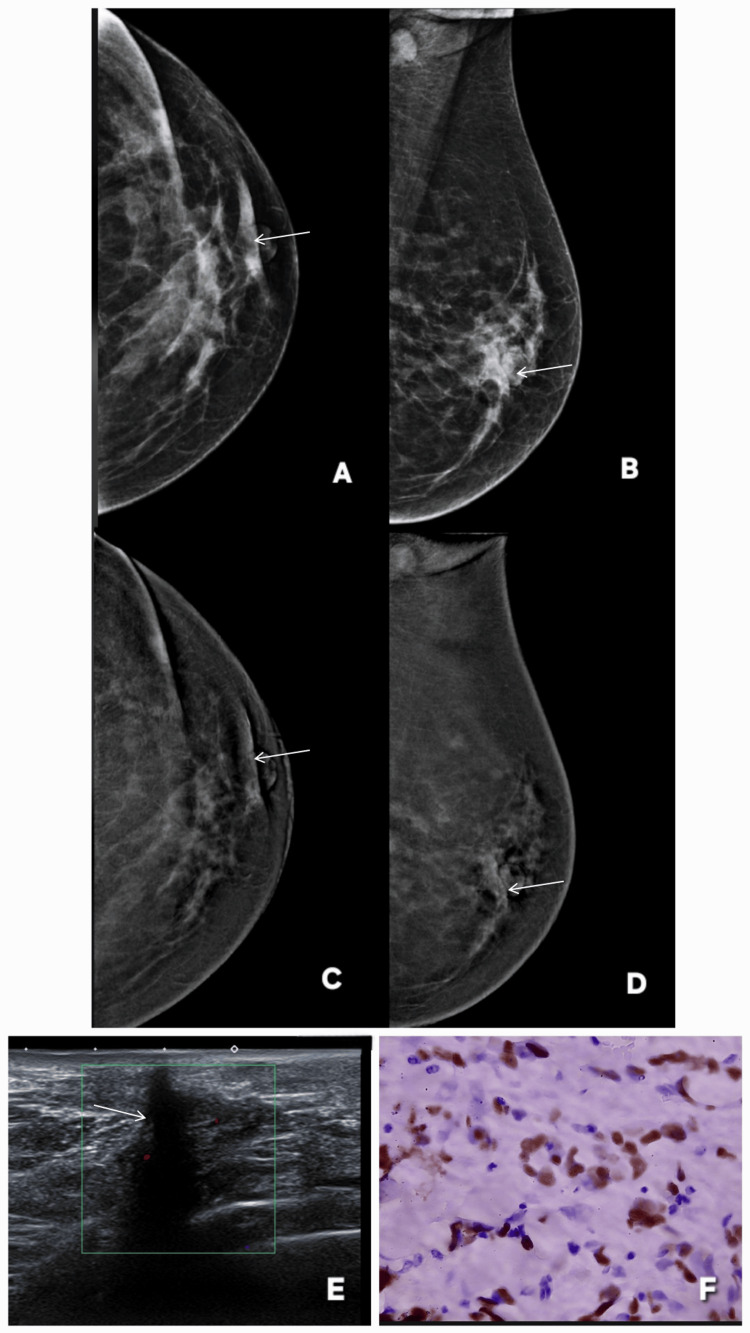
Composite image demonstrating Paget's disease of the nipple in a 59-year-old female patient with corresponding mammographic, ultrasonographic, and immunohistochemical findings as indicated by the white arrows. (A and B) Mammography: Mediolateral oblique and craniocaudal views of the left breast reveal skin thickening of areola with nipple retraction, and an associated irregular, high-density mass with spiculated margins in the retroareolar location. There is associated architectural distortion and no definite calcifications, features suspicious for malignancy (BI-RADS 5). (C & D) Contrast-enhanced mammography recombined images shows enhancing skin thickening, and an irregular enhancing mass with intense contrast uptake and spiculated margins in the retroareolar location, corresponding to the lesion seen on mammography and ultrasound. The lesion exhibits heterogeneous internal enhancement with enhancing linear extensions, consistent with angiogenic activity of invasive carcinoma (BI-RADS 5). No additional enhancing foci or multifocal lesions are identified. (E) Gray-scale ultrasound: Targeted breast ultrasound demonstrates an irregular, hypoechoic lesion with angular and spiculated margins, showing posterior acoustic shadowing and taller-than-wide orientation. The lesion corresponds to the mammographic abnormality. (F) Immunohistochemistry – HER2/neu: Immunohistochemical staining for HER2/neu is negative, showing no complete membranous staining in tumor cells (HER2 score 0). Internal controls show appropriate staining. This indicates HER2 negativity, suggesting the tumor is not eligible for anti-HER2 targeted therapy.

**Figure 20:**
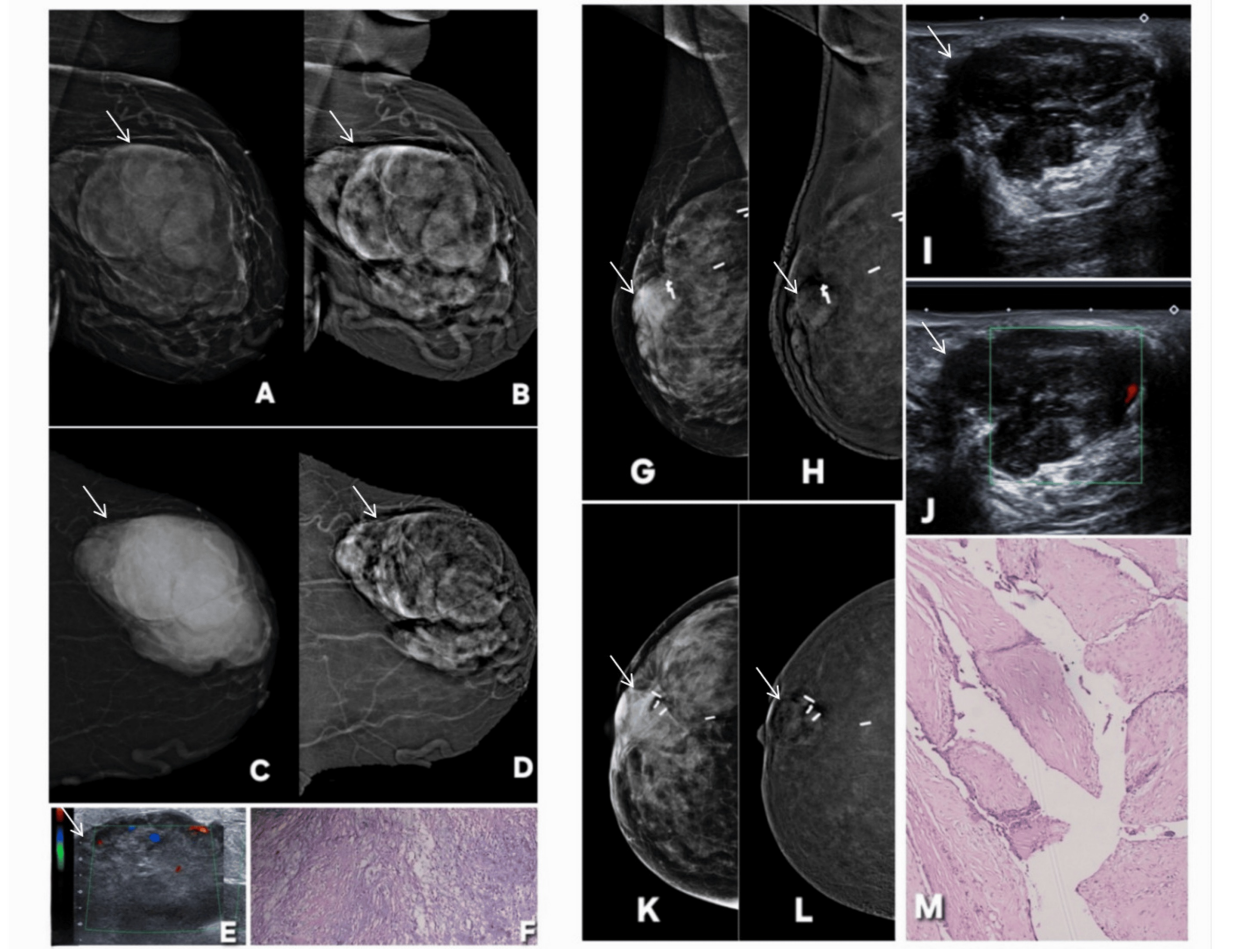
Examples of benign and malignant phyllodes tumor on contrast-enhanced mammography (CEM). Described abnormality is annotated by white arrows in all radiological images. Patient η, a 45-year-old woman. (A, C): Digital mammography (DM) (CC and MLO) shows a large, well-circumscribed lobulated mass in the outer quadrant of the breast (white arrow) (BI-RADS 4). (B, D): CEM views show heterogeneous internal enhancement of the mass (white arrow), with areas of irregular, non-rim enhancement, raising suspicion for malignancy. Margins appear more conspicuous and irregular compared to non-contrast images, reflecting increased vascularity and solid tumor components (BI-RADS 4). (E): Ultrasound of same region shows a heterogeneous, hypoechoic lobulated lesion with internal vascularity on color Doppler, consistent with a hypervascular solid mass. (F): Histopathology confirms diagnosis of malignant phyllodes tumor, showing increased stromal cellularity, nuclear atypia, and stromal overgrowth with leaf-like architecture. Patient ξ, a 43-year-old woman. DM (G: MLO, K: CC) showing postoperative metallic surgical staples in central and upper-outer quadrants of right breast from prior breast-conserving surgery for a benign phyllodes tumor. A new retroareolar, smoothly lobulated soft-tissue density is identified posterior to nipple without associated suspicious calcifications. Based on DM, the lesion was assessed as BI-RADS 4A. CEM (H: MLO, L: CC) show concordant morphology but demonstrate internal enhancement, resulting in an apparent upgrade to BI-RADS 4C on CEM despite the ultimately benign outcome. This enhancement pattern represents a false upgrade caused by the vascular stromal components often seen in phyllodes tumors. Gray-scale ultrasound (I) shows a well-circumscribed, lobulated, heterogeneously hypoechoic mass in retroareolar region, while color Doppler (J) reveals internal vascularity, supporting a solid fibroepithelial lesion. Histopathology (M) confirms diagnosis of benign phyllodes tumor, correlating with imaging features but explaining the false-positive enhancement pattern on CEM. MLO: mediolateral oblique; CC: craniocaudal

**Figure 21:**
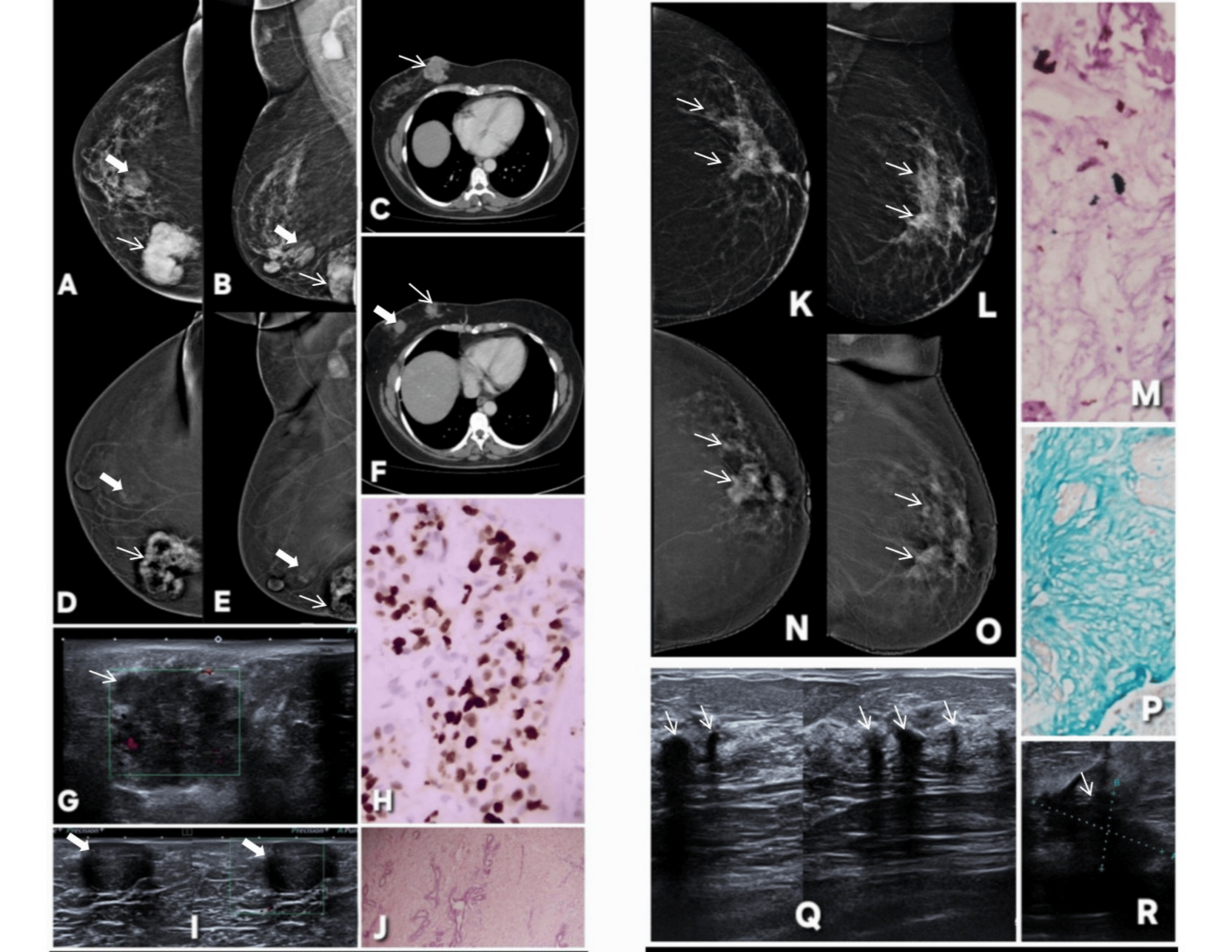
Representative contrast-enhanced mammography (CEM) cases demonstrating concordance between CEM findings and histopathology. The described abnormality is annotated by white arrows in all radiological images. Patient μ (Panels A–J): A 56-year-old female patient with newly developed lump in right breast. Panels A and B show DM of the right breast demonstrating two lesions in the lower inner quadrant. The larger lesion, annotated by a thin white arrow, and the smaller lesion, marked by thick white arrows, are both visualized. Panels D and E show the corresponding CEM images, where the larger lesion demonstrates intense enhancement, whereas the smaller lesion shows minimal enhancement (BI-RADS 4 for larger lesion, BI-RADS 2 for smaller lesion). Panels C and F are corresponding CT images showing the malignant lesion (C) and both lesions together (F). On ultrasound (G and I), the larger lesion appears as an irregular vascular mass (G), while the smaller lesion is a well-circumscribed hypoechoic nodule (I). Histopathological correlation (H and J) confirmed the larger enhancing lesion as invasive ductal carcinoma, which was progesterone receptor positive on immunohistochemistry (H), and the smaller lesion as fibroadenoma (J). These findings demonstrate excellent concordance between CEM enhancement characteristics and histopathological diagnosis. Patient ν (Panels K–R): A 61-year-old female patient with feeling of tightness in left breast. Panels K and L show DM images demonstrating multiple ill-defined lesions (BI-RADS 4). Panels N and O display CEM images showing contrast enhancement within these lesions marked by white arrows (BI-RADS 4). Corresponding ultrasound images (Q and R) demonstrate these lesions as hypoechoic nodules with posterior shadowing. Histopathological evaluation (M) and immunohistochemical staining (P) confirmed the diagnosis of metastatic adenocarcinoma. The imaging findings again show strong concordance between CEM and histopathology in detecting metastatic lesions. DM: digital mammography; BI-RADS: Breast Imaging Reporting and Data System

**Figure 22:**
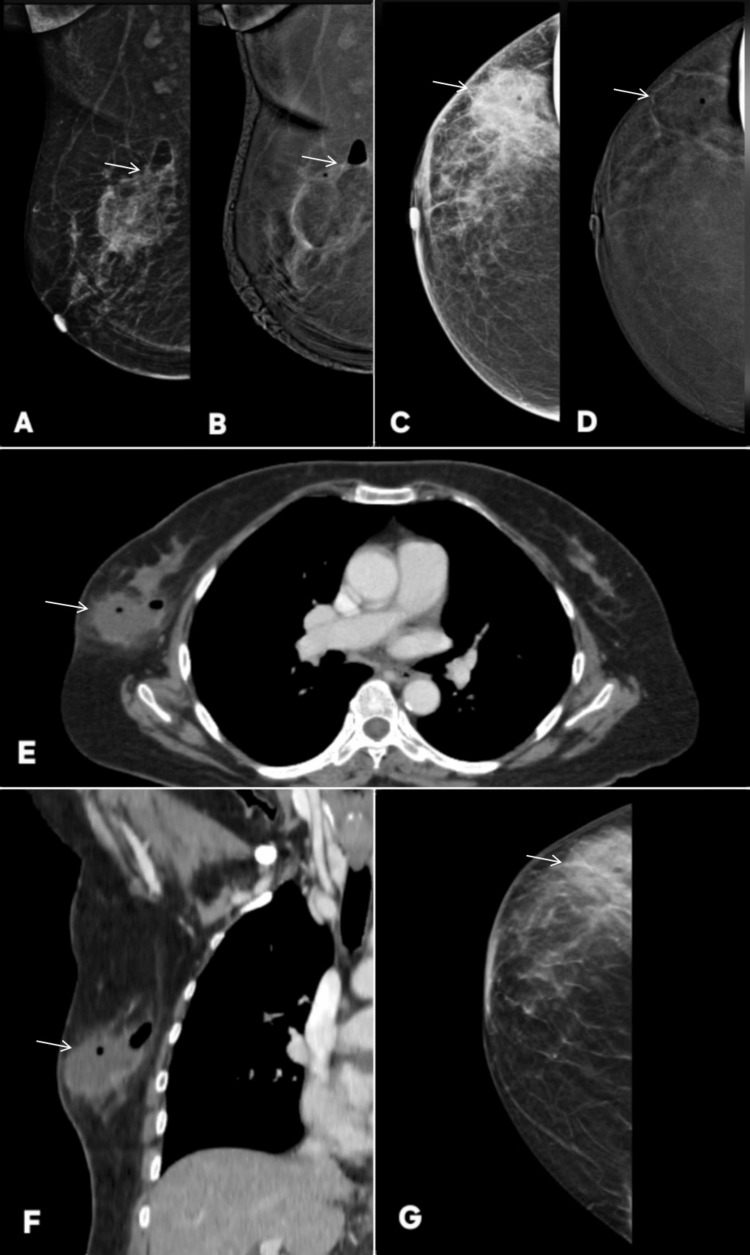
Post-biopsy appearance in a 59-year-old female patient demonstrated on digital mammography (DM), contrast-enhanced mammography (CEM), CT, and digital breast tomosynthesis (DBT). Lesion is annotated with white arrows in all radiological images. DM (A: MLO; C: CC) demonstrates complex cystic mass with internal heterogeneous density and surrounding architectural distortion in the affected breast. Subtle foci of intralesional air are identified, representing post-biopsy changes and helping differentiate procedure-related artifacts from intrinsic tumor characteristics. Mass appears partially circumscribed with focal indistinct margins, raising concern for an underlying malignant component (BI-RADS 4c). Corresponding CEM (B: MLO; D: CC) shows marginal enhancement along cyst wall with enhancing perilesional architectural distortion, features that elevate the suspicion for an infiltrative process. The enhancement pattern is concordant with the morphological abnormalities on digital mammography, supporting a BI-RADS 4c assessment. CEM highlights the vascularized outer rim and the reactive enhancement of adjacent parenchyma, both of which are typical of post-biopsy inflammatory response but may coexist with malignancy. Contrast-enhanced CT of the chest (E: axial; F: coronal) clearly depicts the corresponding complex cystic lesion with foci of air, confirming post-procedural sequelae. The surrounding parenchymal stranding and architectural distortion match the mammographic findings, providing cross-sectional correlation of the lesion’s composite nature. DBT (G: CC) delineates internal heterogeneity, separating superimposed tissue and confirming both the cystic morphology and distortion of adjacent fibroglandular planes. Tomosynthesis appearance is consistent with recent tissue sampling, demonstrating typical post-biopsy appearance across multiple modalities. Together, these images illustrate how post-biopsy changes such as intralesional air, and reactive architectural distortion manifest across digital mammography, CEM, CT, and DBT, and how CEM reliably reproduces and enhances the underlying structural abnormalities without false up- or downgrading. MLO: mediolateral oblique; CC: craniocaudal

**Figure 23:**
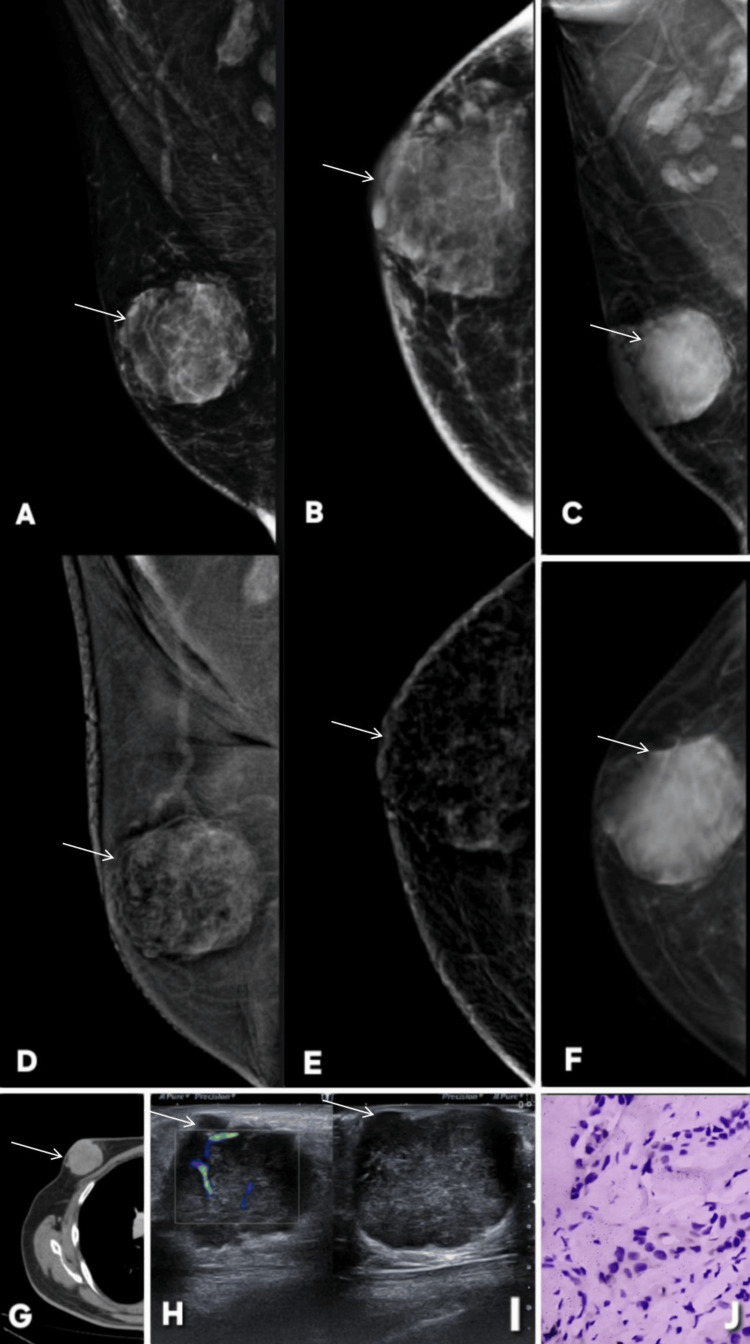
A 64-year-old male patient with breast carcinoma and concordant digital mammography (DM)–contrast-enhanced mammography (CEM) findings. Lesion is marked by white arrows across all radiological images. DM (A: MLO; B: CC) reveals a large, well-circumscribed mass located in the upper outer quadrant of the right breast. The lesion demonstrates mildly lobulated margins with a prominent perifocal lucent halo, a feature frequently associated with malignant infiltration or reactive stromal changes in male breast cancer. Multiple prominent right axillary lymph nodes are also visualized, raising suspicion for nodal involvement (BI-RADS 5). Corresponding CEM (D: MLO; E: CC) shows avid enhancement of the mass, confirming its vascular nature and reinforcing a BI-RADS 5 suspicion for malignancy. The enhancing morphology matches the DM findings with no discordant features. The axillary lymph nodes demonstrate cortical thickening and enhancement, maintaining concordance with DM. Digital breast tomosynthesis (C: MLO; F: CC) further delineates the lesion’s three-dimensional morphology, confirming its lobulated contour and excluding summation artifacts. The adjacent tissue planes appear displaced, consistent with a space-occupying malignant mass. Contrast-enhanced CT of the chest (G, axial) correlates well with mammographic findings, showing an enhancing soft-tissue mass in the upper outer quadrant of the right breast along with enlarged axillary lymph nodes. Color Doppler imaging (H) demonstrates internal vascularity, supporting its solid and likely malignant nature. On targeted ultrasound (I), the lesion appears as a lobulated hypoechoic solid mass with relatively uniform internal echotexture. Histopathology (J) confirms invasive breast carcinoma, and immunohistochemistry demonstrates HER2/neu-negative staining, completing the multimodality correlation. MLO: mediolateral oblique; CC: craniocaudal; BI-RADS: Breast Imaging Reporting and Data System

**Figure 24:**
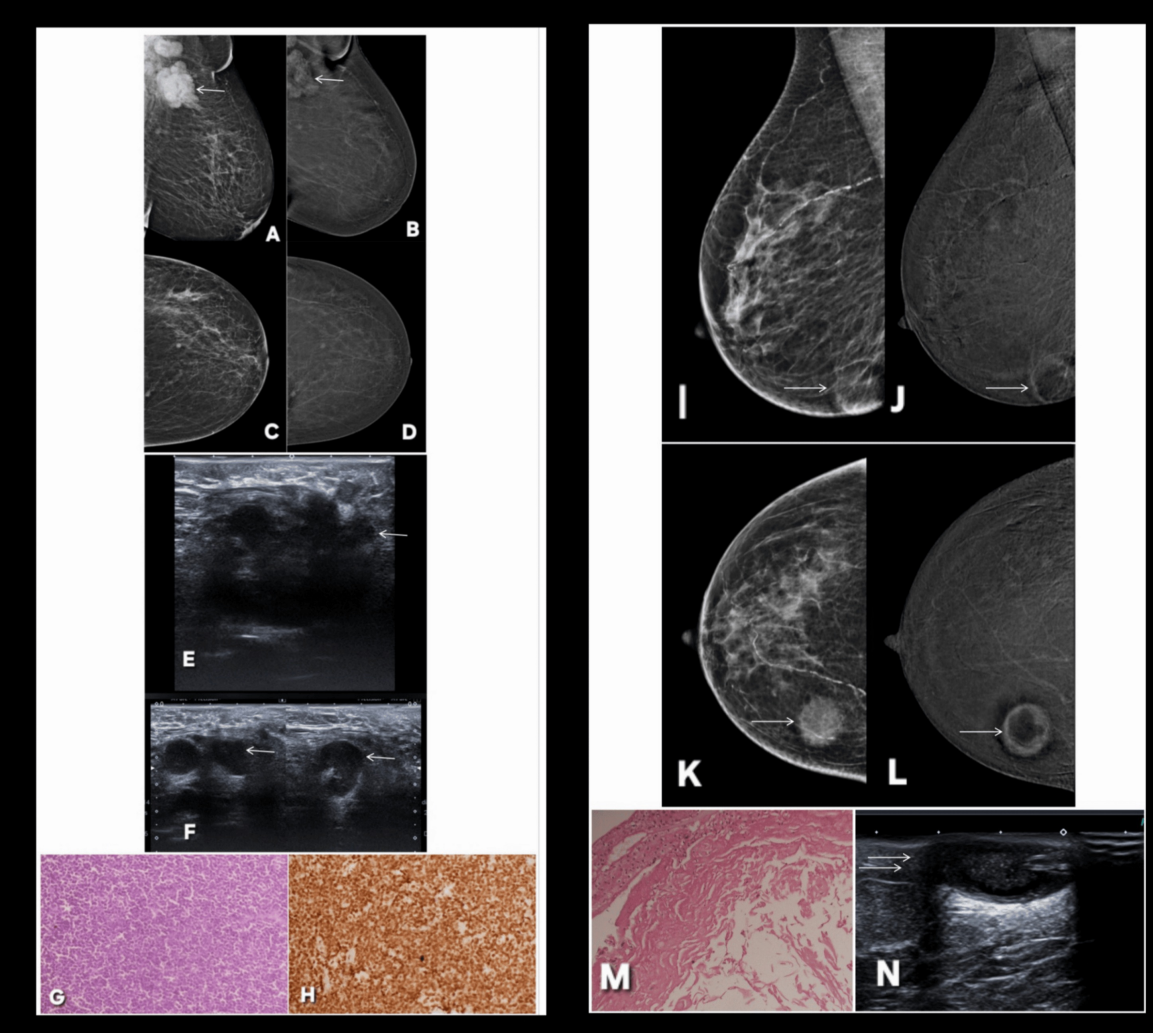
Axillary lymphoma and epidermoid cyst (true negative on contrast-enhanced mammography (CEM) for breast lesion). Lesion is annotated by white arrows in all the radiological images. Patient σ, a 60-year-old woman with lump in left axilla. (A, C): Digital mammography (DM) (CC and MLO) of left breast shows no discrete mass, architectural distortion, or suspicious microcalcifications within breast parenchyma (BI-RADS 1). However, there are enlarged left axillary lymph nodes with rounded morphology and loss of normal fatty hilum (white arrow), raising suspicion for a lymphoproliferative or metastatic process. (B, D): In CEM, the axillary region shows enhancing, enlarged lymph nodes (white arrow), but no corresponding breast parenchymal enhancement is noted, establishing this as a true negative breast finding on CEM. (E, F): Ultrasound of the left axilla show multiple enlarged, rounded lymph nodes with cortical thickening and complete effacement of the fatty hilum (white arrows). No primary lesion is seen in the left breast or axillary tail. (G): Histopathology of lymph node reveals diffuse sheets of atypical lymphoid cells with high nuclear-to-cytoplasmic ratio, vesicular chromatin, and prominent nucleoli, consistent with diffuse large B-cell lymphoma (DLBCL). (H): Immunohistochemistry shows strong CD20 positivity, confirming the B-cell lineage of the neoplasm and establishing the diagnosis of DLBCL. Patient ρ, a 47-year-old woman with breast lump: Example of concordant findings in mammography and CEM. (I, J) MLO views of the breast on DM (I) and CEM (J). (K, L) Corresponding CC views on DM (K) and CEM (L). Both modalities show a well-defined cystic lesion located superficially in the skin, consistent with an epidermoid cyst. The underlying breast parenchyma appears unremarkable. The findings are assigned BI-RADS category 1. (M) Histopathology shows a cyst lined by stratified squamous epithelium with lamellated keratin within the lumen, diagnostic of an epidermoid cyst. (N) Ultrasound demonstrates a well-circumscribed, anechoic lesion with posterior acoustic enhancement confirming its cystic nature. MLO: mediolateral oblique; CC: craniocaudal

## Discussion

Principal findings

This study highlights the significant impact of CEM on refining BI-RADS classification initially based on DM. With correct reclassification or confirmation in nearly 90% of cases, CEM proved highly effective in enhancing lesion conspicuity, especially in dense parenchyma and indeterminate mammograms. Although the study did not directly assess downstream clinical management endpoints such as biopsy avoidance, treatment modification, or altered surveillance intervals, the improved diagnostic classification achieved with the integration of CEM with DM may reasonably be inferred to enhance diagnostic confidence. Such improvements have the potential to influence patient management decisions; however, this impact was not formally evaluated and should be interpreted as an inferred benefit rather than a directly measured outcome.

CEM correctly upgraded 20% of cases, revealing additional malignant foci not appreciated on DM. This aligns with previous reports showing that CEM detects up to 15% additional cancers missed on standard mammography [6].

Comparison with the literature

Several studies have demonstrated that CEM offers sensitivity comparable to breast MRI, with the advantage of lower cost, shorter acquisition time, and easier accessibility. Xiang et al. reported a pooled sensitivity of 97% for both CEM and MRI, with a pooled specificity of 66% for CEM versus 52% for MRI [7]. Pötsch et al. reported a pooled sensitivity for CEM of 91% and for breast MRI 97%, with a pooled specificity of 74% and 69%, respectively [8]. Neeter et al. reported pooled sensitivity values of 96% for CEM and 97% for MRI, with a specificity of 77% for both [9]. Thanh Ha Nguyen et al. found that CEM improved detection of multifocal and multicentric disease [10] while Goh et al. reported CEM influenced surgical planning in 18% of patients [11].

Dromain et al. reported CEM had better diagnostic accuracy than full-field DM alone, or full-field DM and ultrasound [12]. Jochelson et al. found that CEM detected known primary tumors at a rate comparable to MRI [13]. Klang et al. compared CEM with ultrasound for breast cancer detection and showed that CEM has significantly higher accuracy (64% versus 45%) and specificity (40% versus 8%) with a greater sensitivity (97% versus 92%) [14]. Sogani et al. noted that CEM was particularly valuable for problem-solving after inconclusive screening mammography [15]. CEM, hence, plays a role in reducing the number of unnecessary MRI referrals.

In our study, CEM effectively reduced diagnostic ambiguity in BI-RADS 0 and 3 categories by clarifying enhancement characteristics, thereby decreasing the need for further imaging or short-term follow-up.

False-positive and false-negative findings

False-positive enhancement (11.7%) occurred mainly in fibroadenomas and adenosis, reflecting the physiological vascularity of some benign lesions. Awareness of these patterns is essential to prevent overdiagnosis. Previous studies have also reported false-positive enhancement at CEM in infection, inflammation, and benign tumors [16-18].

False-negative downgrades (3.3%) occurred in low-grade or hypovascular invasive ductal carcinoma, emphasizing that the absence of enhancement does not always exclude malignancy, especially in necrotic or treated tumors. Previous studies have also reported false negative findings in CEM [16]. 

Interpretation of agreement

The substantial inter-observer agreement observed for BI-RADS categorization and the excellent reliability for lesion size measurements support the reproducibility of CEM interpretation in clinical practice. These findings reinforce the robustness of the diagnostic reclassification observed in this study and suggest that the reported performance metrics are not solely reader-dependent.

Clinical implications

CEM is a valuable adjunct for dense breasts where DM sensitivity is reduced, BI-RADS 0-3 lesions requiring further clarification, and post-surgical and post-therapy assessments, where it can accurately confirm the absence of recurrence. By improving both sensitivity and specificity, CEM may serve as a bridge modality between DM and MRI, particularly in resource-limited or high-volume clinical settings.

Limitations

This study has several limitations. It is a single-centre study with a modest sample size, which may limit generalizability. Postoperative and post-biopsy changes in some patients may have influenced lesion appearance and contributed to false upgrades on CEM. Long-term follow-up for benign lesions was limited, and comparison with contrast-enhanced breast MRI, the current functional imaging standard, or cost-effectiveness analysis was not performed. There is a potential selection bias due to the inclusion of only symptomatic patients, which may limit generalizability to screening populations. The diagnostic accuracy metrics were also calculated on a per-patient basis rather than a per-lesion basis. Although this approach avoids statistical dependence when multiple lesions are present in the same patient, it may underestimate the full impact of CEM in patients with multifocal or multicentric disease. Future studies with larger cohorts should incorporate lesion-level analyses using appropriate statistical models to better characterize diagnostic performance. The inclusion of lesions confirmed by imaging follow-up rather than histopathology may introduce verification bias, as imaging stability does not provide the same level of diagnostic certainty as tissue diagnosis. Lastly, although inter-observer agreement was assessed and demonstrated substantial to excellent reliability, interpretation variability remains an inherent limitation of imaging-based studies.

## Conclusions

CEM substantially refines the BI-RADS classification initially assigned on DM by revealing additional malignant lesions, confirming benign findings, and resolving indeterminate interpretations. In this study, CEM correctly upgraded, downgraded, or confirmed findings in 88.3% of the cases, demonstrating strong concordance with histopathology. While false-positive and false-negative results exist, CEM remains a reliable, rapid, and cost-effective tool that enhances diagnostic confidence, particularly in dense breasts and equivocal mammograms. Integration of CEM into diagnostic workflows can significantly optimize patient triage, reduce unnecessary biopsies, and improve overall breast cancer detection accuracy. While these findings suggest potential clinical value, the impact on downstream patient management requires confirmation in prospective studies incorporating management and outcome endpoints.
